# Engaging African American Youth in the Development of a Serious Mobile Game for Sexual Health Education: Mixed Methods Study

**DOI:** 10.2196/16254

**Published:** 2020-01-31

**Authors:** Loral Patchen, Lindsey Ellis, Tony Xuyen Ma, Corilyn Ott, Katie H K Chang, Brook Araya, Sravanthi Atreyapurapu, Amal Alyusuf, Robin Gaines Lanzi

**Affiliations:** 1 MedStar Washington Hospital Center Washington, DC United States; 2 Benten Technologies Manassas, VA United States; 3 University of Alabama at Birmingham Birmingham, AL United States

**Keywords:** sexual health, sex education, African Americans, youth, adolescents, video games, games, experimental, community-based participatory research, user-centered design

## Abstract

**Background:**

Although teen pregnancy rates decreased dramatically in the United States over the past decade, the rates of sexually transmitted infections (STIs) among adolescents and young adults increased. STI rates disproportionately affect African American youth and young adults. Innovative, accessible, and culturally relevant sexual health interventions are urgently needed.

**Objective:**

This study aimed to identify the optimal modality for a game-based sexual health intervention; develop the educational, entertainment, and technological aspects of the serious game; and demonstrate its usability and acceptance by the target population.

**Methods:**

This project was grounded in formative data collection with community-based participatory research principles and practices combined with a user-centered design and development approach. Sexually Active Adolescent–Focused Education (SAAFE) was developed using input and feedback from African American youths aged 15 to 21 years who participated in a youth advisory board and focus group discussions to inform the co-design and cocreation of the serious game. The process was highly iterative with multiple sessions for user input following design changes. It proceeded in 3 stages. Social cognitive theory and problem-solving theory were leveraged to provide evidence-based, trauma-informed education through a serious game. Usability testing assessed the quality of user experience with the prototype.

**Results:**

Across all 3 stages, a total of 86 self-identified African American males and females aged 15 to 21 years from the District of Columbia and Birmingham, Alabama, participated. Participants requested a dating simulation game. They wanted SAAFE to be customizable, realistic, entertaining, educational, modern, and experiential, linking consequences to their gameplay decisions. Usability testing resulted in an initial System Usability Survey score of 77.7, placing the game in the 82nd percentile and above average for usability.

**Conclusions:**

Initial results suggest that the SAAFE prototype is a promising intervention to engage African American youth in sexual health education using a role-playing game. If proven efficacious, the game has the potential to meet the need for sex education, counterbalance unhealthy portrayals of sex in popular media, and respond to the disparities in the STI epidemic.

## Introduction

### Background

The sexual health of adolescents and young adults in the United States remains a public health crisis. Youths aged 15 to 24 years acquire half of all new sexually transmitted infections (STIs), yet many do not obtain screening [1]. A 2013 survey revealed that only 27% and 9.8% of 15- to 25-year-old sexually experienced females and males, respectively, had been tested for STIs in the past 12 months [2]. Significant disparities in sexual health also exist among the youth. African Americans make up 13% of the US population, but in 2017, they accounted for 27.7% of new chlamydia infections, 31.8% of new syphilis infections, 39.7% of new gonorrhea infections, and 43% of new HIV infections [1,3]. African American high school students are significantly more likely than other race and ethnic groups to identify 4 or more sexual partners within their lifetime [4].

Improving the sexual health of adolescents and young adults and reducing racial disparities are public health priorities included in Healthy People 2020. However, analysis of the Center for Disease Control and Prevention’s National Survey of Family Growth found that receipt of formal sex education is both inadequate and on the decline in the United States. Between 2011 and 2013, although approximately three-quarters of teens received education on STIs and HIV, less than three-quarters were taught how to say no to sex and only half received education on condoms [5]. New approaches to the delivery of sexual health content that is engaging to the youth and culturally specific are needed.

Serious video games are increasingly used for health education purposes and show promise as educational tools [6]. A serious video game is defined as “an interactive computer application, with or without a significant hardware component, that has a challenging goal, is fun to play with, incorporates some concept of scoring, and imparts in the user a skill, knowledge or attitude which can be applied in the real world” [7]. The main difference between a serious video game and a video game in general is that the former is developed for pedagogical purposes beyond the entertainment and recreational goals of the latter [8].

Young Americans, regardless of race or ethnicity, are prolific video game players [9]. About 90% of teens and 67% of young adults report playing video games, and about a quarter of teens believe that they spend *too much time* on these games [10-12]. African Americans are more likely to view video games positively (ie, promote teamwork and communication and problem-solving and strategic thinking skills), which may reflect greater engagement among this group [13]. Video games can be played on a mobile device (such as a phone or a tablet), a gaming console, or a computer, but African American youth are more likely to depend on their mobile devices to access video games on the Web [14].

Sexual health is an emerging focus for serious video games, and the results are promising [15]. A good example is *PlayForward: Elm City Stories* developed by the Play2Prevent Lab at the Yale Center for Health & Learning Games. It showed promise in improving sexual health knowledge and skills among youths of color aged 11 to 14 [16]. Another example is *It’s Your Game* developed by the University of Texas Prevention Research Center. *It’s Your Game* is a computer-based sexual health education game designed for middle schoolers of any race and ethnicity in a classroom setting; it demonstrated effectiveness in delaying initiation of sexual activity [17-19]. However, no comprehensive sexual health game has been developed *with* older African American adolescents and young adults for use on a mobile platform.

### Objectives

This study describes the development of a mobile-based serious video game for sexual health education, Sexually Active Adolescent–Focused Education (SAAFE), designed specifically with and for youths who identify as African American. The goal of our intervention is to promote healthy sexual behaviors that, among other things, help reduce the disproportionate burden of STIs among African American youth.

Before launching intervention development, our multidisciplinary scientific team convened a panel with expertise in clinical service delivery of adolescent sexual health, developmental psychology, and mobile health (mHealth) technology. The purpose of the panel was to assist our team with content development, align the project with existing research, and identify behavioral targets of interest.

Social cognitive theory (SCT) and problem-solving theory (PST) provided theoretical frameworks for design and development decisions. SCT describes the multiple, reciprocal influences on health behaviors, including individual experiences, beliefs, and environmental factors [20]. SCT states that “knowledge of health risks and benefits of different health practices, perceived self-efficacy, outcome expectations about the expected costs and benefits, health goals people set for themselves, and perceived facilitators and social and structural impediments” can translate knowledge into effective health practices [20]. Building self-efficacy (confidence in one’s ability to perform a desired health behavior) and self-regulation (goal setting and planning) are key constructs of SCT that guided the development of SAAFE. PST describes a *problem-solving* process whereby an individual “gains new pieces of information and finds out gradually which circumstances affect, or don’t affect the removal of the cause, the conflict or the difficulty” [21].

This paper describes the scope and process of our work, presents findings, describes the research leading to the final serious game, shares lessons learned, and outlines plans to proceed to the next phase: an outcome evaluation; gaming optimization; and, if impact is demonstrated, widespread implementation.

## Methods

### Overview

A community-based participatory research approach, integrated with user-centered design and development methods, effectively informed the development of SAAFE. The process was highly iterative, with multiple sessions for user input following design changes. It proceeded in 3 stages, described below. Across all stages, a total of 86 self-identified African American males and females aged 15 to 21 years from the District of Columbia (DC) and Birmingham, Alabama (AL), participated.

**Figure 1:**
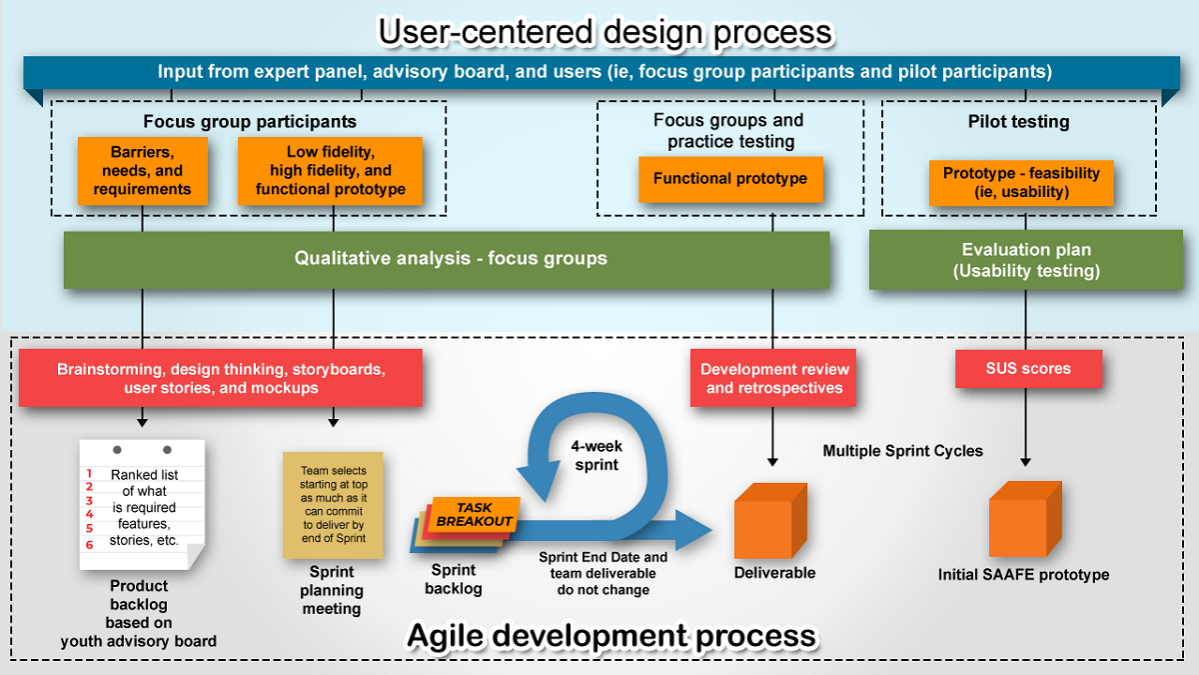
User-centered design processes. SAAFE: Sexually Active Adolescent–Focused Education; SUS: System Usability Scale.

First, a youth advisory board provided input to develop a game prototype. This prototype was then tested for usability. Subsequently, we conducted a series of focus groups to further develop and finalize the game. We adopted an Agile methodology, which is an iterative process whereby the game is built incrementally in biweekly development cycles, called *sprints*, that allows for user feedback after each cycle. Editing and developing the game between focus groups allowed participants to systematically review our progress. Figure 1 shows an illustration of this framework for SAAFE development.

This study was approved by the Institutional Review Boards of the MedStar Health Research Institute and the University of Alabama at Birmingham.

### Participants

African American youths aged 15 to 21 years were included in the study. Females who were pregnant or within 6 weeks post-partum were excluded because attitudes and behaviors during the prenatal and immediate postpartum period may be systematically different. Written informed consent was obtained for participants aged 18 years or older; parental consent with the adolescent’s assent was obtained for participants younger than 18 years.

Initially, 6 African American youths were recruited from the DC area to participate in the youth advisory board and inform the initial prototype development. Participants of the youth advisory board responded to a description of the work made available at nonprofit organizations focused on the youth and an adolescent health center. A study team member met with the interested youths to explain the project, review eligibility criteria, and obtain consent and assent to participate.

New participants then tested the SAAFE game prototype and completed a usability survey. Recruitment occurred at an adolescent health center in DC over 4 weeks. Prospective participants were informed about the study by a health center team member. If interested, they were asked to complete a short questionnaire to verify eligibility as described above, and then, the participants signed an informed consent document. They were then provided access to the game.

Usability data were obtained for the prototype to ensure acceptability. Results were promising, so the team held a series of focus groups with new participants to guide additional development. To capture additional geographic variability in youth perspectives, we expanded our sample to include Birmingham, AL. Like DC, AL has a high STI burden. Participants were recruited from adolescent health and community centers to participate in focus groups over the course of 9 months. Many youths took part in 2 or more focus groups. Each focus group lasted about 2 hours and included 6 to 10 youths. Two groups ran concurrently and reviewed the same topics in AL; 1 group included youths who identified as lesbian, gay, bisexual, transgender, queer/questioning, intersex, or asexual (LGBTQIA) and the second group included youths in which LGBTQIA status was not specified. The LGBTQIA status of the DC groups was not specified. Focus group discussions were held in private rooms and were led by a trained focus group moderator. Participants were asked to sign an agreement to keep the identity of others confidential. They received a US $50 cash incentive for participating in each focus group.

### Game Development Process

The project began with a review of the scientific literature and consultation with a panel of experts in the fields of adolescent sexual health, child development, and mHealth intervention strategies to define outcome parameters and behavioral health targets. We then recruited the youth advisory board to inform initial design decisions and prototype development. Gaming experts with graduate and undergraduate degrees programmed the game with assistance from graphic artists, script writers, and audio/visual specialists.

#### Youth Advisory Board

Guided by our design and development framework of maximizing engagement of community users, the initial design and features of the game were driven by 4 meetings with the youth advisory board that took place in DC over the course of 8 months with 2 months between the first 3 meetings and 4 months between third and fourth meetings. An overview of the topics discussed is presented in Textbox 1. The initial SAAFE game prototype—an initial version of the game—was developed and refined based on feedback obtained after each meeting.

**Overview of the topics explored with the youth advisory board.**

Meeting 1

- Introduction
- Video game use habits
- Sexual health topics
- Sexual language identification

Meeting 2

- Game concept
- Storyline, character, and scene design

Meeting 3

- Storyboard design
- Game navigation

Meeting 4

- Game play
- Feedback on graphics and game content

The resulting prototype was then shared with the youth advisory board. They were given tablets with the game preinstalled. Members reviewed the game instructions and provided feedback on clarity, language, length, and ease of understanding. They played the prototype for at least an hour and provided feedback on content, artwork and design, minigames, dialogue, and entertainment value. Meeting facilitators recorded the session and developed a list of key recommendations from members. The design team listened to the recording and used the key recommendations list to inform the final refinements before usability testing.

#### Usability Testing

Youths participating in usability testing were provided a tablet with the game preinstalled and a unique user code. They played the game for an hour and then completed the System Usability Scale (SUS), a highly reliable (Cronbach alpha of .91) 10-item Likert scale that measures the user’s experience with an electronic tool [22]. Specifically, SUS measures function, efficiency, effectiveness, and satisfaction. It is the most common questionnaire used in assessing system usability. Possible scores range from 0 (not usable) to 100 (perfectly usable). A score of 68 is considered average, so a score above 68 implies that a tool has greater than average usability. We asked 4 additional questions with a 1 to 5 scale (strongly disagree to strongly agree): “I would consider downloading the SAAFE game to play,” “I learn a lot about STIs using the SAAFE game,” “I would recommend the SAAFE game to a friend,” and “Overall, I am satisfied with playing the SAAFE game.” The goal was to achieve an average score of 4 or higher.

#### Focus Groups

Usability metrics for the SAAFE prototype were promising, so we proceeded to conduct focus groups, which were an integral part of our user-centered, highly iterative process of cocreation. Focus group participants reviewed content and design, and we modified the game based on those findings. We used Microsoft PowerPoint and YouTube videos to aid in discussions of features and scenarios. We discussed dialogue with printouts given to each participant. We provided tablets with the game preinstalled for gameplay. Feedback from each focus group was analyzed, compared and contrasted, and incorporated into the game before the next focus group. Table 1 provides an overview of the topics explored in each focus group.

**Table 1:** Overview of focus group discussion topics.

Focus group number	Topic	Sample questions
Focus groups 1 and 2	General feedback and features	What are your general impressions? How do you feel about the game’s destinations and setting, characters and interactions, multiplayer capability, minigames, and education? What are the most important changes we should make? What ideas do you have for other features?
Focus groups 3 and 4	Scenarios	Review of storylines and possible outcomes Are the scenarios realistic? What else would you like to see happen?
Focus groups 5-7	Dialogue	Line-by-line dialogue review Does the dialogue resonate with you? What would you change?
Focus group 8 (District of Columbia only)	General feedback and gameplay	Extended gameplay What are your general impressions? What do you like about the game? What do you not like? Would you play this game?

Each focus group session was audio-recorded and transcribed verbatim, omitting all identifying data. Transcribed documents were downloaded onto a password-protected computer for data analyses. NVivo 11 was used to begin the open coding process and the hierarchical coping model–guided analysis as themes emerged. Findings were shared with the game development team throughout the process to inform game development and refinements.

## Results

### Participants

In total, 86 youths identifying as African American aged between 15 and 21 years participated in this study. Overall, 6 individuals, 3 males and 3 females, participated in the youth advisory board. One female member withdrew because of school and work conflicts after the first session. Finally, 26 youths participated in usability testing, and 54 individuals, 23 from AL and 31 from DC, participated in focus groups. A total of 8 focus groups were held in DC, 7 were held in AL with the LGBTQIA group, and an additional 7 were held in AL with the LGBTQIA status-unspecified group.

Regarding focus group participants, half of the participants (27/54) identified as male and 30% (16/54) identified as LGBTQIA. Most participants, 67% (36/54), were engaged in some kind of employment, and 65% (35/54) of the participants were in high school. Moreover, 35% (19/54) of the participants reported never playing video games, whereas 22% (12/54) of the participants reported playing games daily. Two-thirds of the participants (36/54) reported a history of sexual activity. Among participants with a history of sexual activity, 64% (23/36) reported using a condom during their most recent sexual encounter and 72% (26/36) had been tested for STIs at least once. Complete demographics and descriptive information are available in Table 2.

**Table 2:** Demographics, gaming habits, and sexual history of focus group participants.

Participant response		Value, n (%)^a^
**Sex**		
	Male	27 (50)
	Female	26 (48)
	Trans female	1 (2)
**Age (years)**		
	15	5 (9)
	16	7 (13)
	17	10 (19)
	18	14 (26)
	19	6 (11)
	20	8 (15)
	21	4 (7)
**Identify as lesbian, gay, bisexual, transgender, queer/questioning, intersex, or asexual**		
	Yes	16 (29)
	No	36 (67)
	NR^b^	2 (4)
**School status**		
	High school	35 (65)
	College	18 (33)
	Not in school	1 (2)
**Employed**		
	Yes	36 (67)
	No	18 (33)
**Videogame play frequency**		
	Never	19 (35)
	1-2 days per week	12 (22)
	3-4 days per week	7 (13)
	5-6 days per week	3 (6)
	Everyday	12 (22)
	NR	1 (2)
**Ever sexually active**		
	Yes	36 (67)
	No	15 (28)
	NR	3 (6)
**If yes, age at first sex (years)**		
	<11	1 (3)
	11	3 (8)
	13	4 (11)
	14	6 (17)
	15	8 (22)
	16	6 (17)
	17	4 (11)
	18	2 (6)
	NR	2 (6)
**Condom use during last sex**		
	Yes	23 (64)
	No	11 (31)
	NR	2 (6)
**Ever used condoms**		
	Yes	32 (89)
	No	2 (6)
	NR	2 (6)
**Ever tested for STIs^c^**		
	Yes	26 (72)
	No	9 (25)
	NR	1 (3)
**Ever had an STI**		
	Yes	5 (14)
	No	29 (81)
	NR	2 (6)
**Number of sex partners now**		
	0	9 (25)
	1	22 (61)
	2	1 (3)
	4	2 (6)
**Number of sex partners in the last month**		
	0	5 (14)
	1	20 (56)
	2	3 (8)
	4	2 (6)
	5	2 (6)
	>5	1 (3)
	NR	3 (8)
**Number of sex partners in the last year**		
	0	1 (3)
	1	14 (39)
	2	6 (17)
	3	3 (8)
	4	4 (11)
	5	2 (6)
	>5	1 (3)
	NR	5 (14)

^a^Some percentages do not add up to 100 because of rounding.

^b^NR: not reported.

^c^STI: sexually transmitted infection.

### Youth Advisory Board: Initial Prototype Development

Participants in the youth advisory board informed the design and development of the prototype SAAFE game. The main contributions of the youth advisory board are described below.

A *dating simulation game* was the preferred game concept. The research team presented 3 other choices to the youth advisory board: an American Idol or rap storybook, a professional athlete role play, and Game of Life board game. Members quickly and unanimously expressed preference for the dating simulation game. They were energized about creating their own character and making decisions through simulated real-life situations. The research team also presented minigames to embed within the game to enhance player engagement. Members liked all the games, which included *STI Shooter*, *Condom Run*, and *Fact Pop*.

Youth advisory board members wanted *characters that were realistic and customizable*. When asked about character design, they did not want cartoons and preferred characters that reflected their own ages. Characters should have different body types and skin tones, with many options for customization through clothing, tattoos, and accessories.

Members wanted *realistic settings, situations, and dialogue.* They preferred an urban setting and rejected settings they interpreted as *rich* or that seemed too perfect. The research team introduced options for situations and dialogue, and again, they wanted it to be as *real* as possible, often rejecting attempts at humor and preferring language they use in their everyday lives.

Youth advisory board members recommended that *instructions be part of game play*. Initial game instructions were tedious and too long and used words unfamiliar to them. Members would skip past them and *figure it out* as they played. They also pointed out that the meaning of status bars was unclear. For example, if the fatigue bar was full, members were not sure if their character was tired or well rested.

Members recommended *more sexual health education* in the game. They enjoyed game play but expected to learn more. They recommended that the pop-ups and dialogues deliver more information.

All youth advisory board feedback was incorporated before initiating usability testing of the SAAFE prototype.

### Usability Testing of the Initial Prototype

According to a review of usability studies, 20 participants are sufficient to identify at least 95% of existing usability problems for a prototype [23]. Overall, 26 participants completed the SAAFE prototype usability testing and 23 completed the SUS survey. SAAFE’s average SUS score was 77.7, which is higher than the average SUS score of 68 [24]. A SUS score of 80.3 or higher would place a game in the top 10% with regard to usability and is the point at which a user would recommend a game to a friend [25]. This benchmark score is within SAAFE’s average score margin of error.

In addition, 25 of the 26 users responded to the 4 additional questions and agreed or strongly agreed that “I would recommend the SAAFE game to a friend” and “Overall, I am satisfied with playing the SAAFE game,” with mean scores of 4.4 and 4.32, respectively. “I would consider downloading the SAAFE game to play” had an average score of 3.92, and “I learn a lot about STIs using the SAAFE game” had an average score of 3.96. These scores approached the goal score of 4, indicating that participants on average agreed with these statements or felt neutral.

### Focus Group Findings: Game Development

The promising usability findings for the SAFFE prototype justified additional game development. Focus groups yielded the following key insights: offer extensive opportunities for avatar customization, including sexual orientation; create realistic and relatable storyboards; strike a balance between education and entertainment; use current technology that is as sophisticated as other games; allow youths to experiment and experience the consequences of their actions through storyboards; and include elements of competition and game-play incentives. A summary of the main themes is provided in Table 3.

**Table 3:** Themes emerging from focus groups regarding the game development.

Theme and its description		Representative quotes
**Realistic**		
	Participants wanted the game to resemble the real world as much as possible	“The whole point is to educate about what type of things go in your real life. It needs to be realistic.” “I feel like it’s a small world right there.” “If you could add like a moving like...moving items...like a car driving around...add movement and to make it realistic...Maybe a bird or a plane...in the distance that will be good.”
**Relatable**		
	Participants wanted to relate to the language, situations, and characters	“Also may be like adding stuff about like the LGBT community...LGBTQ community as well cuz...it seems very heterosexual in there.” “You should be able to pick the person who you want to be and not be just the generic.” “This is just an idea...But if he gave people the option to customize their own avatar basically...they’re going to want to do it cuz it’s like it’s them...they can be who they want and they can let...you know they can learn more...But if you give them a random person...they will be like I didn’t want that person...if it is a dude...a 30 year old girl will be like I don’t want a dude...I am not dude.” “I also think some the language that they were using is a little bit like make me wanna roll my eyes a little bit like the way that they were talking to the...the narrator was talking to us like I don’t know I can’t remember what he said exactly but some of the stuff we were like what...I did not feel like authentic.”
**Customizable**		
	Participants wanted the ability to choose the gender, appearance, sexual orientation, and personality of the avatar; the type of community; and the education that they would be exposed to	“If we had a really short hair option for girls.” “I just like for at least there to be a lot of options for dark skin.” “I think that when you have that at such a young age, they’re not going to think anything of it and I think it is more likely to reinforce that dialogue is something that is acceptable.”
**Entertaining**		
	Participants preferred less text, more audio, and more entertainment to maintain engagement	“The information that they were giving were really good...I think that there needs to be a little bit more fun though cuz I think it’s like someone...wouldn’t play." “Even though like it is important information like you can shorten it for fun because it would be bored.” “Ya coz there is a lot of reading seems unnecessary.”
**Experiential**		
	Participants desired to experiment with high-risk behaviors and experience consequences of those behaviors	“I think there should be a little bit of a scare in the game.” “If you get pregnant, it’s just like game over.” “In real life, you can’t go back, but in this game, you can learn.” “If you die you’re going to be mad, you’re going to be mad, but it’s like you know you did that Victory Royale...you will be like I did it...I got it.”
**Modern technology**		
	Participants reported that they are accustomed to having many high-quality, high-functionality videogame options and would be unlikely to endure a game that was perceived to be outdated or difficult to use	“You know video games are like really realistic now and so if you’re trying to get [early teens] to want to pay attention to it, you’re going to want something that’s going to catch their eyes. If it is something like [school], they are going to just keep tapping until it is over.” “Because you don’t, you don’t want to make this whole game and go through this whole hassle and people are just tapping tapping and getting these coins and it’s all for nothing.”

*Customization* was a frequent topic of conversation and a high priority for focus group participants. Participants wanted their game avatars to better represent themselves. They wanted to choose the gender, appearance, clothing, sexual orientation, and personality of their avatar. Focus group participants specifically recommended offering a variety of skin tones, facial features, hair colors and styles, clothes, head coverings, jewelry, and props. Participants that identified as LGBTQIA requested gender-neutral options and elimination of binary options. Customizing the community—urban or suburban and high income or low income—was also suggested. Nearly all participants responded well to location-specific information about sexual health sites where they could get STI testing or free condoms, among other services. In addition, there was a discussion about customization of content for different age groups. For example, many older adolescent participants wanted to see vivid pictures of STIs but thought that might not be appropriate for younger teens. They suggested different game versions for different age groups.

Achieving a *realistic and relatable* game was a high priority for participants across focus groups. According to participants, the virtual world in the first version of the game looked like a *rich neighborhood* and unlike their own urban neighborhoods. They also expected the health center to look more like an emergency room, which was perceived to be a more routine place to obtain health care. The virtual world also needed more locations. Suggested locations included a restaurant, store, mall, friend’s house, hotel, place of employment, library, school or college campus, dorm room, sports arena, movie theater, concert, club, bar, house party, and sex party.

Language, situational relevance, and dialogue proved to be highly nuanced and variable depending on the geographic location. For example, the slang term *lit* (meaning *exciting* or *excellent*) was agreed upon in both locations. *Mumbo* sauce (a condiment) is most popular in DC and raised questions in Birmingham. Both groups felt that baking a cake was not relatable, so it was swapped with making a pizza. In addition, early focus groups felt that dialogue with nonplayer characters was too short and that sexual proposals were too abrupt. Participants wanted real-life relationship building to be reflected in the game, which was further developed with dialogue and expanded storylines. In addition, 1 group discussed the importance of using language that discusses sex and sexual behavior in a way that is a matter of fact, positive, and not shaming. Some felt that the game assumed emotional intimacy in sexual relationships, although in reality, that is not always the case.

The appeal for *incentives and competition* in the game was nearly universal. All groups recommended that storylines include rewards for safe, healthy behaviors and consequences for high-risk behaviors. They suggested the ability to make and spend money within the game, perhaps for avatar enhancements such as a greater range of hairstyles. Some participants wanted to invite and compete with other players and to share their scores on social media. Minigames were offered as a way to earn and use *currency*. The minigames are sexual health themed and intended to be entertaining more than educational so that players stay engaged. An arcade was added featuring a range of minigame options to keep the players engaged and to earn money for use in the game.

Focus groups universally desired to *experiment* with high-risk behaviors and experience the sometimes unpleasant and difficult consequences of those behaviors. According to the youth, experiencing harsh consequences of risky behaviors in the virtual world would facilitate learning that could be applied to the real world. It would enhance the game’s realism as well. One group suggested poor appearance or death as an outcome if you drink too much or do not get STI treatment. Participants were eager to replay a scenario so that they could choose a different behavior and see how the outcomes changed.

The youths expected the SAAFE technology, functionality, and graphics to be *modern and sophisticated.* They reported that they are accustomed to having many high-quality, high-functionality videogame options and would be unlikely to endure a game that was perceived to be outdated or difficult to use. They expected character animations—such as walking and dancing—and graphics to be more realistic and novel. One participant requested “maybe a bird or a plane in the distance.” Nearly all participants expected music and audio features.

## Discussion

### Principal Findings

The main findings of this study were that a sample of African American youth in DC and AL preferred a dating simulation game that allowed experimentation with high-risk behaviors and that was customizable, entertaining, educational, realistic and relatable, incentivized and competitive, and modern and sophisticated. The SAAFE game was developed in partnership with the youth, and the youth found the SAAFE game to be highly usable.

### Sexually Active Adolescent–Focused Education Game

Findings from focus groups led to the refinement and development of the final SAAFE game described here. The SAAFE game centers on visiting different locations in the virtual world and interacting with other nonplayer characters to build relationships and have intimate encounters. In response to focus group findings, 7 sexual health–themed storylines, or *missions*, were developed. Each mission represents a content area with learning objectives. Missions can be played sequentially or randomly. The first storyline *New Kid on the Block* introduces the player to the game and discusses the use of condoms and dental dams. The second storyline *Getting Tested* centers on STIs: transmission, health impacts, symptomology, testing modalities, and treatment, and a health care visit is modeled. *Making PrEParations* discusses the transmission, natural history, and treatment of HIV/AIDS and the use and benefits of pre-exposure prophylaxis (PrEP) in preventing HIV/AIDS. Storyline 4 *Just Say Yes or No* teaches about sexual assault, coercion, and incapacity and models sexual consent. Storyline 5 *Use It* centers on condom negotiation. Storyline 6 *All Romance* simulates relationship building and healthy communication about sex. The final storyline *Sex, Drugs, and Rock N’ Roll* reviews the impact of substance use on sexual behaviors. Subthemes that run across multiple storylines include behaviors that mediate STI risk; abstinence and limiting sex partners; accessing health care; and responsible technology use, including sexting and pornography.

**Figure 2:**
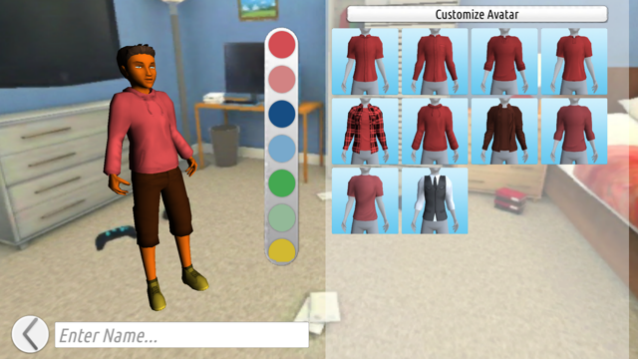
Screenshot of character design.

**Figure 3:**
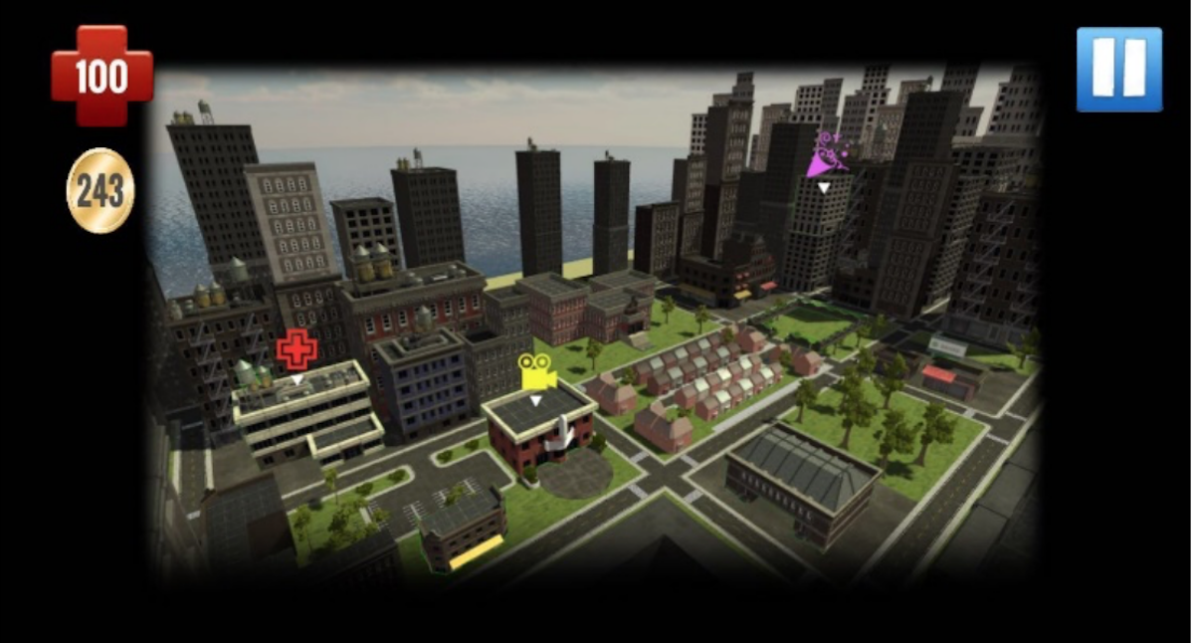
Screenshot of Studenton city map.

**Figure 4:**
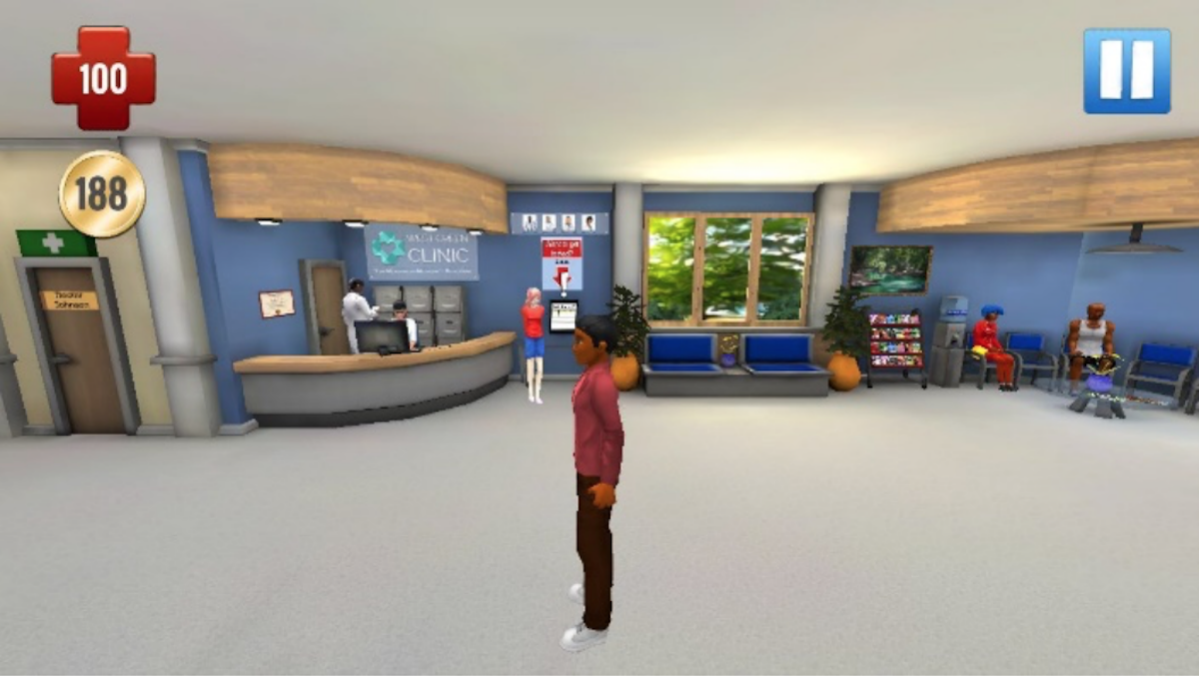
Screenshot of the clinic.

**Figure 5:**
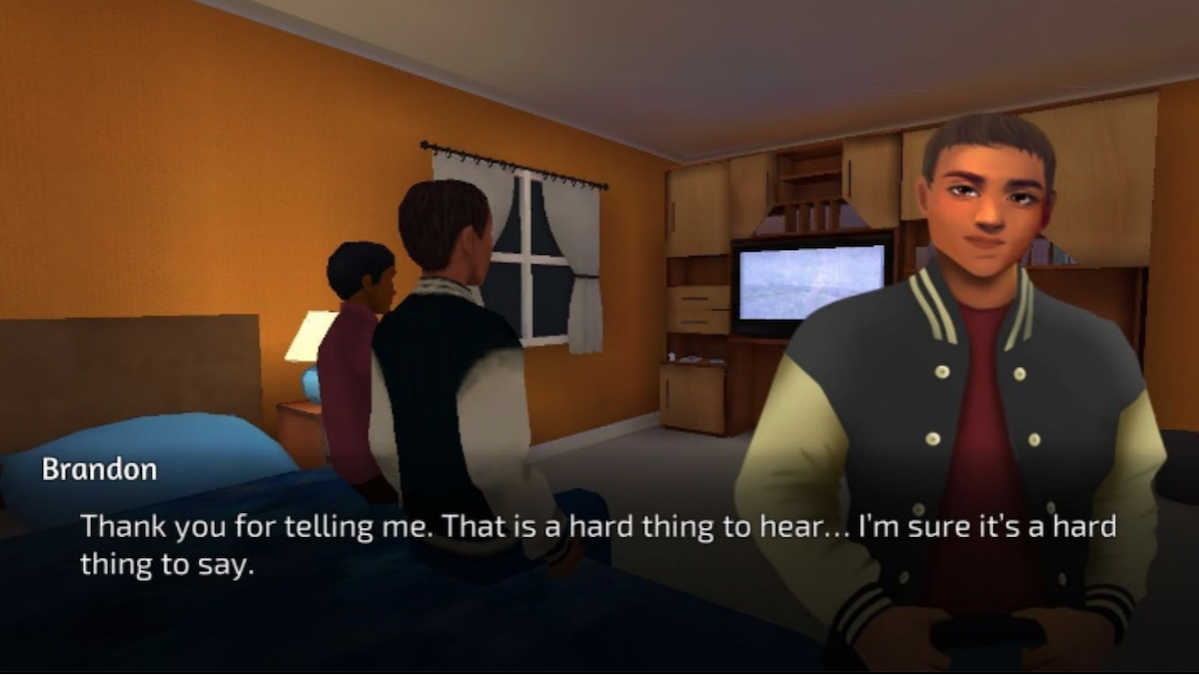
Screenshot of dialogue with a nonplayer character at home.

Findings emphasize the importance of players having the ability to customize their avatar’s appearance and sexual orientation (see Figure 2 for a screenshot of character design). To improve the delivery of game-play instructions, we followed focus group guidance and oriented players to game features as part of the first mission (see Figure 3 for a screenshot of the Studenton city map). Game settings were developed based on focus group suggestions, including different school locations (cafeteria, homeroom, and sex education classroom) and city locations (movie theater, clinic, gym, store, video arcade, house party, and home; see Figure 4). As players interact with nonplayer characters, they are asked to select responses and behaviors that become increasingly sexually intimate and potentially high risk. As desired by our participants, healthy behaviors and learning are rewarded with coins and increases in health and attractiveness and relationship meters, whereas unhealthy behaviors are discouraged through decreases in the same meters. Undesirable outcomes such as STI acquisition, breakups, and loss of friends also occur in response to unhealthy behaviors (see Figure 5 for a screenshot of dialogue with a nonplayer character at home).

**Figure 6:**
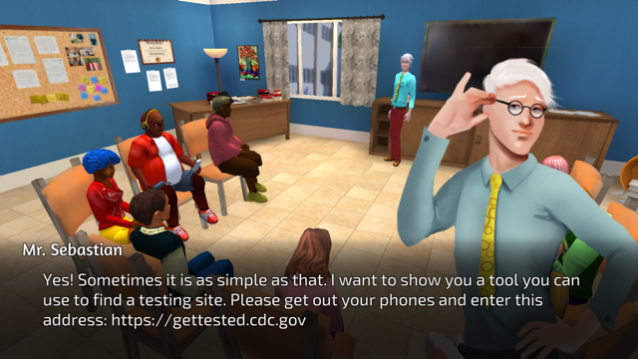
Screenshot of sex education class.

**Figure 7:**
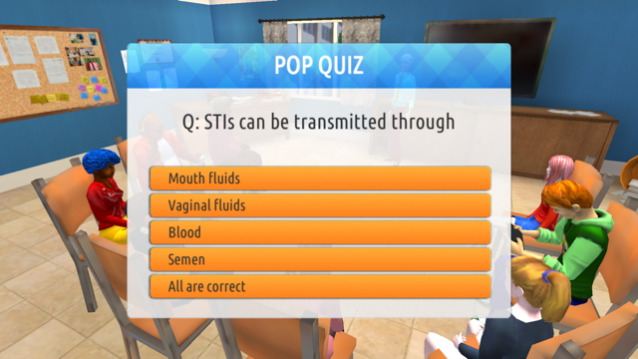
Screenshot of a pop quiz.

**Figure 8:**
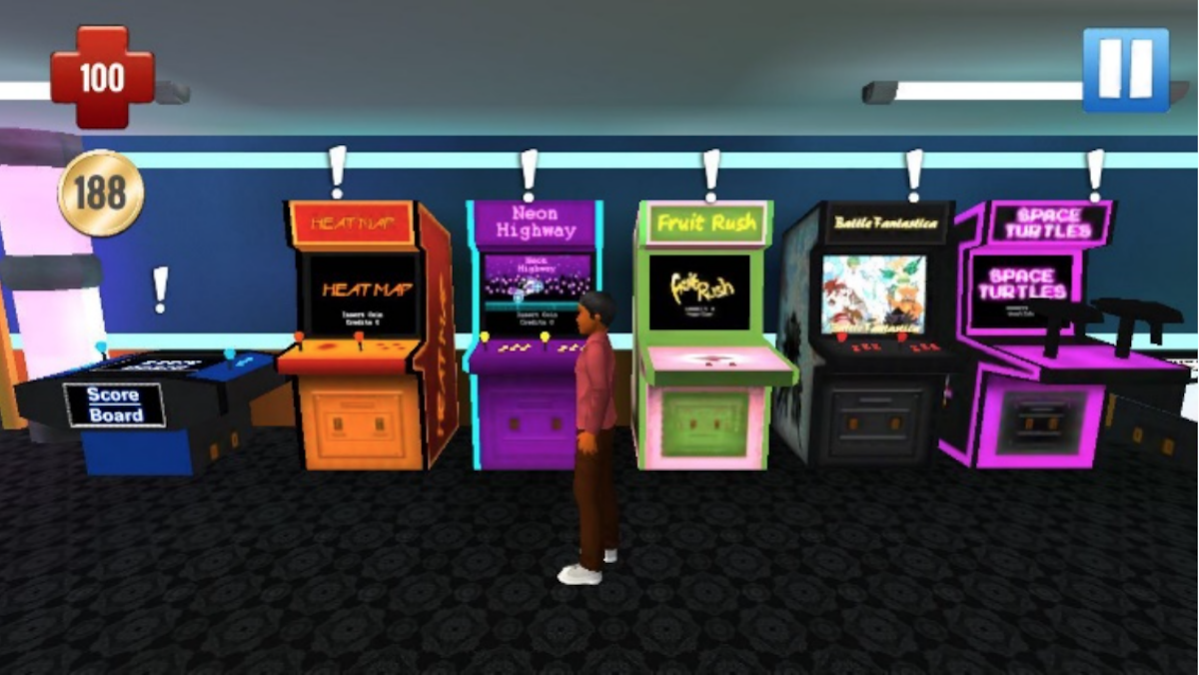
Screenshot of the arcade.

Delivery of educational content is consistent with focus group preferences. Players attend sex education class and converse with elders for more traditional didactic information exchange (see Figure 6). They can elect to view posters and videos that are located throughout Studenton. Pop quizzes, pop-ups, and links to outside resources are also scattered throughout the game (see Figure 7), and the arcade includes a sexual health trivia minigame (see Figure 8). Learning and skill building also occur as players move through the storylines and experience the rewards or consequences of their actions.

The mobile elements of SAAFE provide opportunities to customize content to local communities and specific audiences. For example, a link to or listing of local resources that provide sexual health care services and free condoms can be embedded. The legal status of sexual health services—for example, the age at which youths can consent to STI testing without parental involvement—varies geographically, and this information may be customized in the game. Videos and infographics embedded in the game can be swapped to meet the educational needs of specific audiences. This feature allows SAAFE to be useful and valuable to a multitude of entities, such as schools and health centers, and across geographic locations.

Creating and fostering community-engaged research with African American youth was critical to the successful development of the SAAFE game. The initial high usability index was a direct result of the partnership with the youth to ensure the game was not only relatable but also fun. This study has further elucidated how community-based participatory research can enable a transfer of knowledge and skills between the community and academic scientists. Indeed, youths participating in this project were highly engaged and committed and contributed sophisticated and nuanced insight. Adopting an Agile game development methodology also proved invaluable. Editing and developing the game between focus groups allowed participants to systematically review our progress. This interactive approach allowed the design team to collaborate effectively and ensure SAAFE appropriately reflected the feedback from focus groups.

Regarding theoretical frameworks, SAAFE applies SCT and PST by presenting information, modeling behaviors, and allowing players to experiment with behaviors that lead to desirable and undesirable outcomes. The youth are provided with engaging and educational pop-ups, quizzes, videos, and links to outside resources. Avatar-based realistic scenarios task youths with making sexual health decisions, and they virtually experience the outcomes of their decisions. Unhealthy behaviors may result in STIs, pregnancy, or the end of relationships. Ideally, players will develop self-efficacy, self-regulation, and problem-solving skills with regard to sexual health behaviors that will translate to the identification and adoption of those behaviors in real life.

### Challenges and Lessons Learned

The sexual exposures and learning needs of youths aged 15 to 21 years are quite different. The information was novel, yet sometimes overwhelming, for younger youths, whereas older youths appreciated more detail and nuance but were already familiar with much of the information. Greater age specificity may be beneficial. Technology today is sophisticated, and the youth have many game and entertainment choices. Keeping pace with their high expectations and competing to win their attention will continue to be a challenge as long as a gap remains in research and development of commercial entertainment and serious game products.

Striking the right balance between education and entertainment was complex. Youths will engage more meaningfully with the game if they are entertained, yet the game is intended to be educational, and the youth expect to learn something. They expressed frustration with an imbalance either way. The game in its current form does not contain all educational content that we would have liked, most notably, contraception. Our team plans to develop reproductive health themes with next steps and additional resources.

Developing a game that is both customizable to the user while also being relatable and relevant to the larger demographic group proved challenging. We learned that it was easier to develop dialogue and content regarding facts. It was harder to develop dialogue and content to cultivate intimate communication skills and obtaining sexual consent. Dialogue, in particular, required many iterations. Geographic variations in language, culture, and access to health care led the team to adopt more generic language and information at the cost of specificity. The option to customize the game to different regions of the United States via a built-in dialogue engine mitigates this concern.

Thoughtful use of language was key. It was important to discuss sex and sexual behaviors in a way that is trauma informed, direct, nonjudgmental, and nonshaming. Youths also preferred language that was familiar and realistic. The development team desired language that was scientifically accurate and attentive to the psychological and emotional impact of words. This was perhaps the most complex aspect of developing SAAFE.

By modeling behaviors and conversations that are healthy and respectful, this game is part of a solution to contemporary social problems such as sexual assault and marginalization of persons living with HIV. We endeavored to counter stereotypes and include groups that have historically been excluded, including women, people of color, and LGBTQIA individuals. Participants in this project defied stereotypes that the youth are ill informed, reckless, and thrill seeking. On the contrary, youths desire to be responsible and healthy. The final game ensures to represent the youth in a manner that is accurate and respectful.

### Implications

SAAFE is a highly usable mobile game that effectively engages African American youth to learn about and simulate healthy sexual behaviors. Further research, in the form of a larger randomized control trial, is currently underway to evaluate the game’s ability to deliver health education, promote condom utilization, and prompt the youth to seek STI testing and treatment. Secondary outcomes of interest include PrEP knowledge and sexual consent knowledge, attitudes, and behaviors. Future versions will also include comprehensive contraception content.

Our Agile and community-based participatory research methodologies can be applied to the development of other serious games, particularly those of the simulation or role-play genres. Our results and lessons learned are also generalizable to serious game developers.

The initial findings suggest that SAAFE has the potential to help meet the need for innovative approaches to sex education and that SAAFE offers an opportunity to augment more traditional didactic sexual health education in an effort to promote healthy sexual behaviors and reduce the burden of STIs among African American youth.

## Appendix

Multimedia Appendix 1 Powerpoint presentation of study highlights with screenshots.

## Abbreviations

AL: Alabama
DC: District of Columbia
LGBTQIA: lesbian, gay, bisexual, transgender, queer/questioning, intersex, or asexual
mHealth: mobile health
PrEP: pre-exposure prophylaxis
PST: problem-solving theory
SAAFE: Sexually Active Adolescent–Focused Education
SCT: social cognitive theory
STI: sexually transmitted infection
SUS: System Usability Scale
