# Supplementary material for: Engaging African American Youth in the Development of a Serious Mobile Game for Sexual Health Education: Mixed Methods Study
Source: JMIR Serious Games. 2020 Jan 31;8(1):e16254. doi: 10.2196/16254 (PMC7055799; doi:10.2196/16254)

## Slide 1
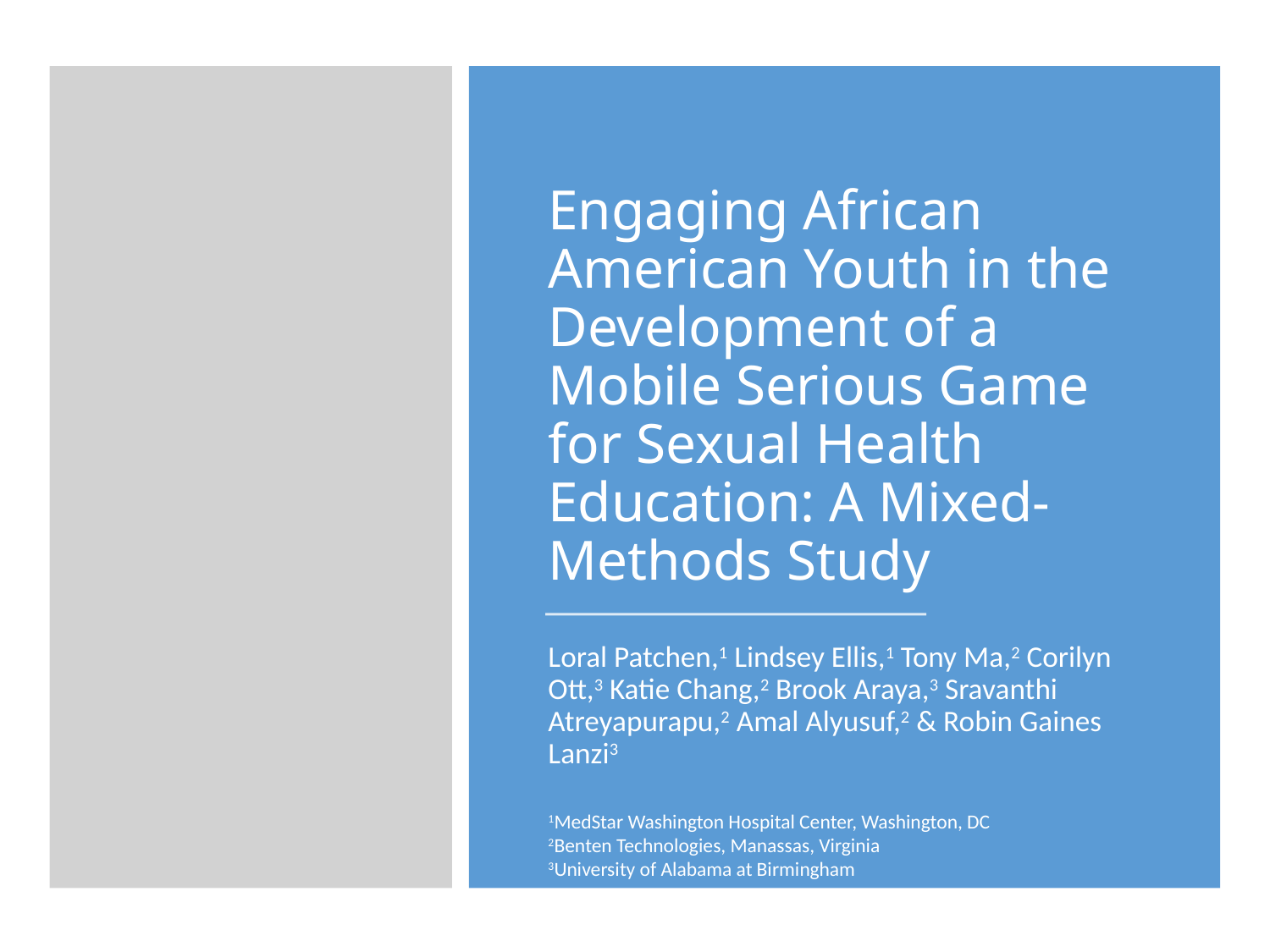

# Engaging African American Youth in the Development of a Mobile Serious Game for Sexual Health Education: A Mixed-Methods Study
Loral Patchen,1 Lindsey Ellis,1 Tony Ma,2 Corilyn Ott,3 Katie Chang,2 Brook Araya,3 Sravanthi Atreyapurapu,2 Amal Alyusuf,2 & Robin Gaines Lanzi3
1MedStar Washington Hospital Center, Washington, DC
2Benten Technologies, Manassas, Virginia
3University of Alabama at Birmingham

## Slide 2
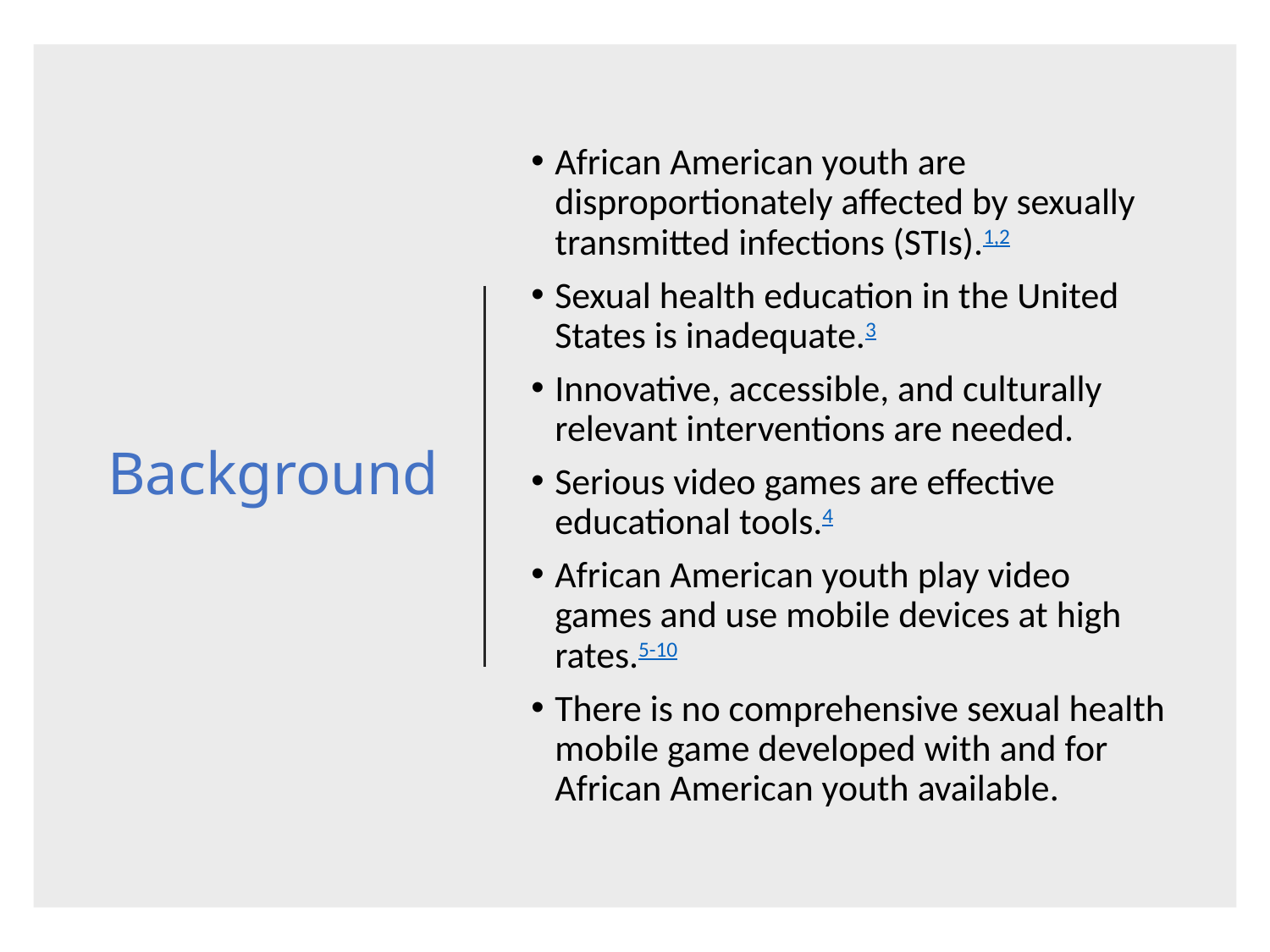

# Background
African American youth are disproportionately affected by sexually transmitted infections (STIs).1,2
Sexual health education in the United States is inadequate.3
Innovative, accessible, and culturally relevant interventions are needed.
Serious video games are effective educational tools.4
African American youth play video games and use mobile devices at high rates.5-10
There is no comprehensive sexual health mobile game developed with and for African American youth available.

## Slide 3
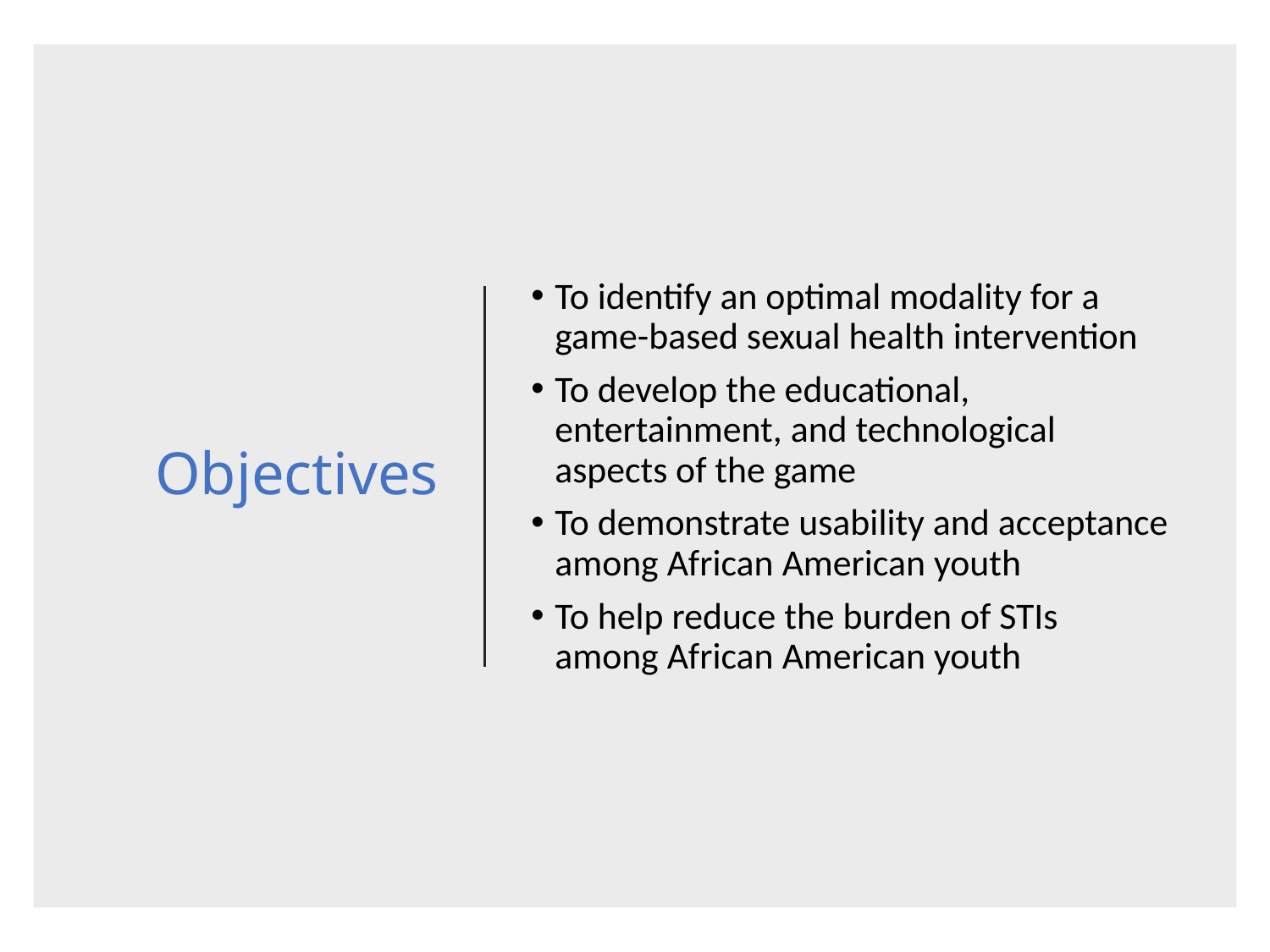

# Objectives
To identify an optimal modality for a game-based sexual health intervention
To develop the educational, entertainment, and technological aspects of the game
To demonstrate usability and acceptance among African American youth
To help reduce the burden of STIs among African American youth

## Slide 4
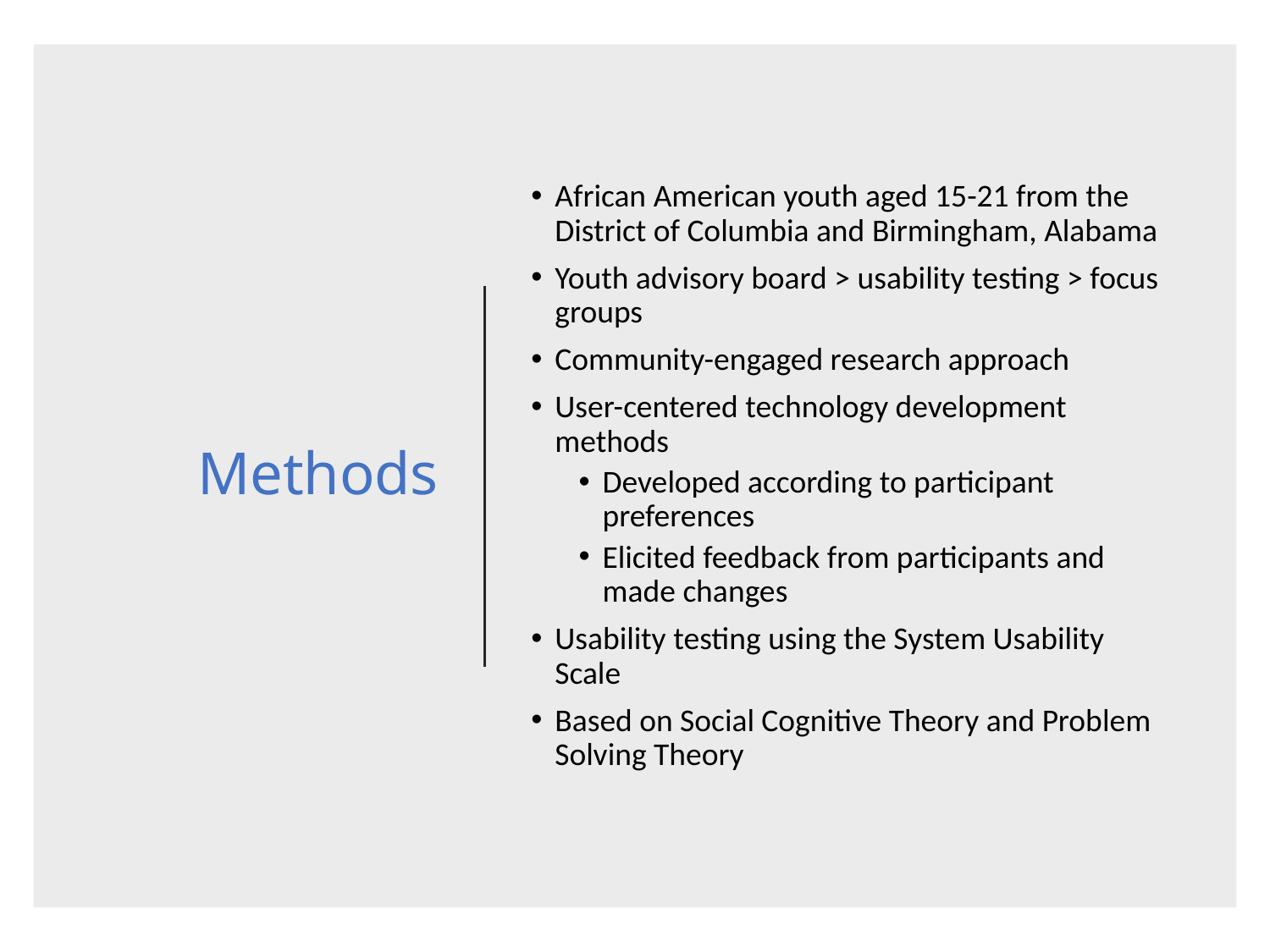

# Methods
African American youth aged 15-21 from the District of Columbia and Birmingham, Alabama
Youth advisory board > usability testing > focus groups
Community-engaged research approach
User-centered technology development methods
Developed according to participant preferences
Elicited feedback from participants and made changes
Usability testing using the System Usability Scale
Based on Social Cognitive Theory and Problem Solving Theory

## Slide 5
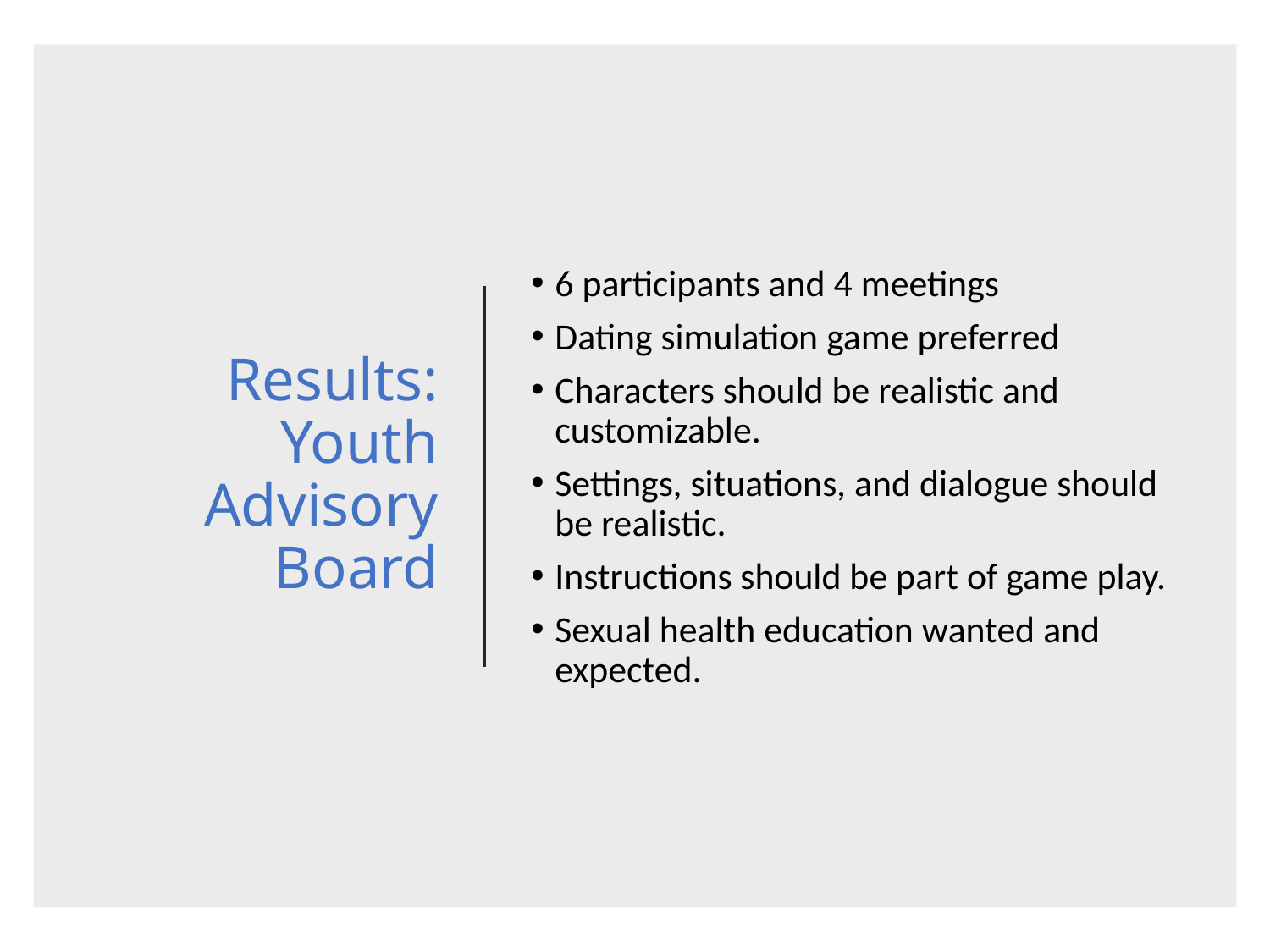

# Results: Youth Advisory Board
6 participants and 4 meetings
Dating simulation game preferred
Characters should be realistic and customizable.
Settings, situations, and dialogue should be realistic.
Instructions should be part of game play.
Sexual health education wanted and expected.

## Slide 6
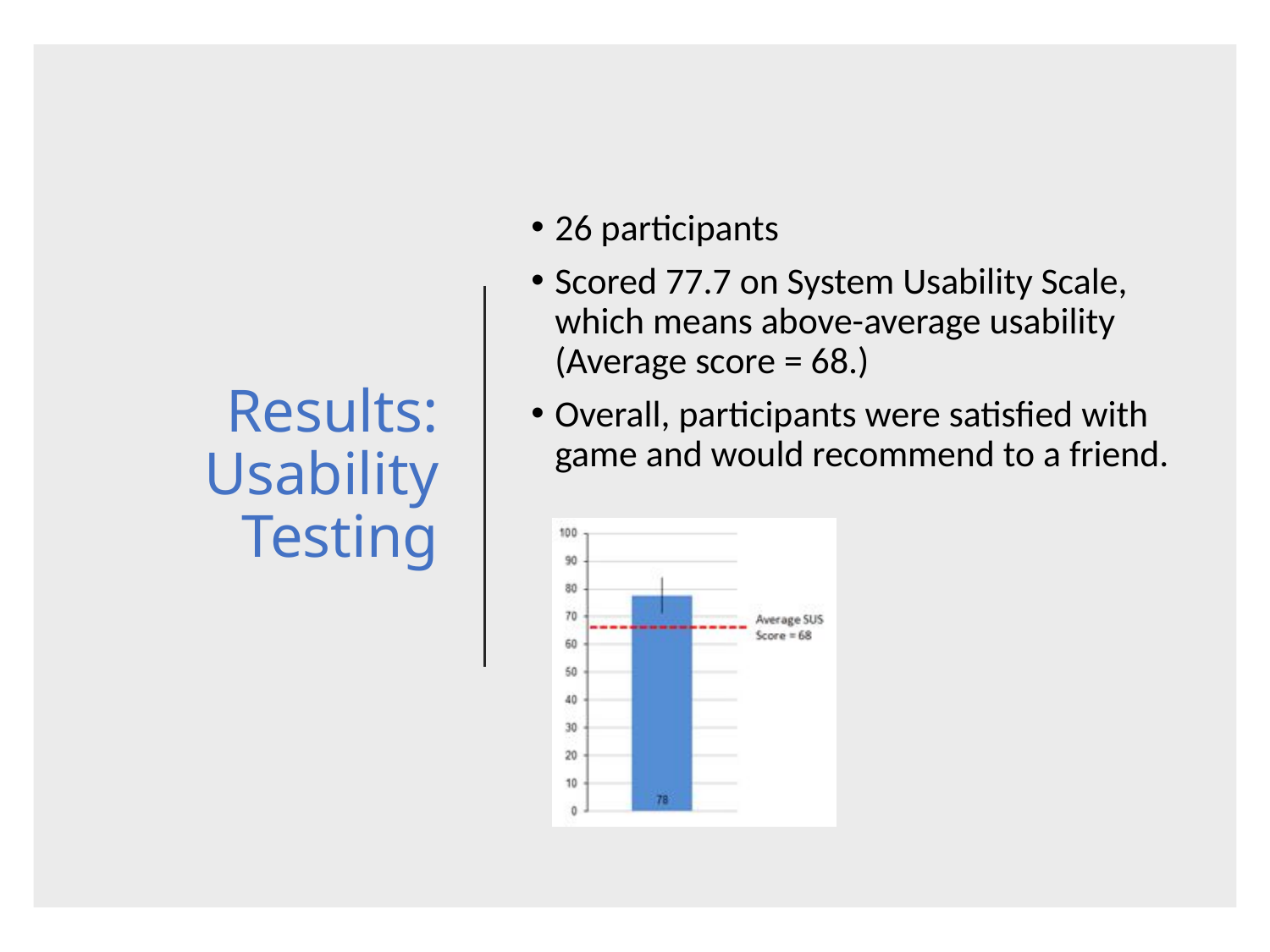

26 participants
Scored 77.7 on System Usability Scale, which means above-average usability (Average score = 68.)
Overall, participants were satisfied with game and would recommend to a friend.
# Results: Usability Testing

## Slide 7
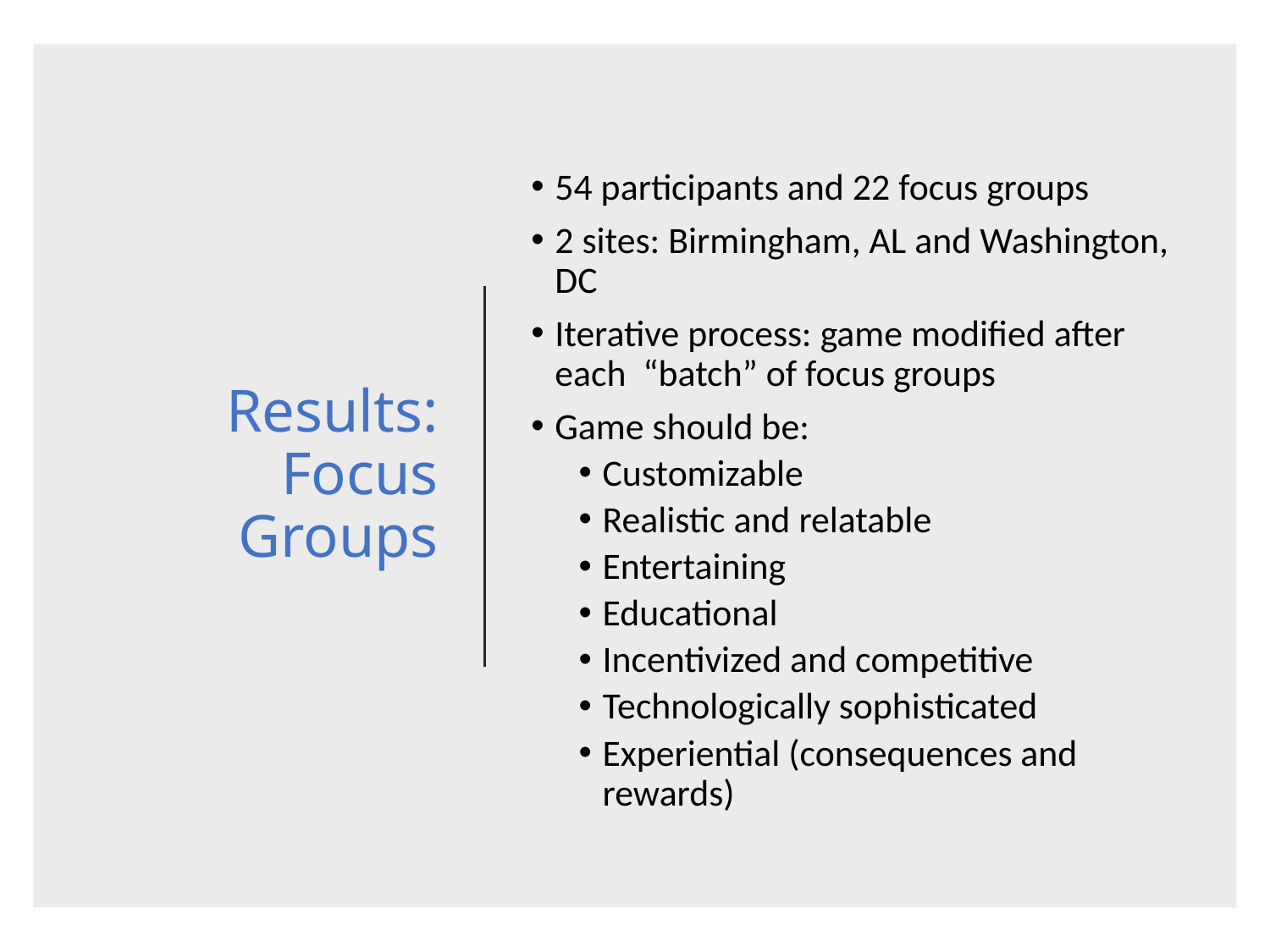

# Results: Focus Groups
54 participants and 22 focus groups
2 sites: Birmingham, AL and Washington, DC
Iterative process: game modified after each “batch” of focus groups
Game should be:
Customizable
Realistic and relatable
Entertaining
Educational
Incentivized and competitive
Technologically sophisticated
Experiential (consequences and rewards)

## Slide 8
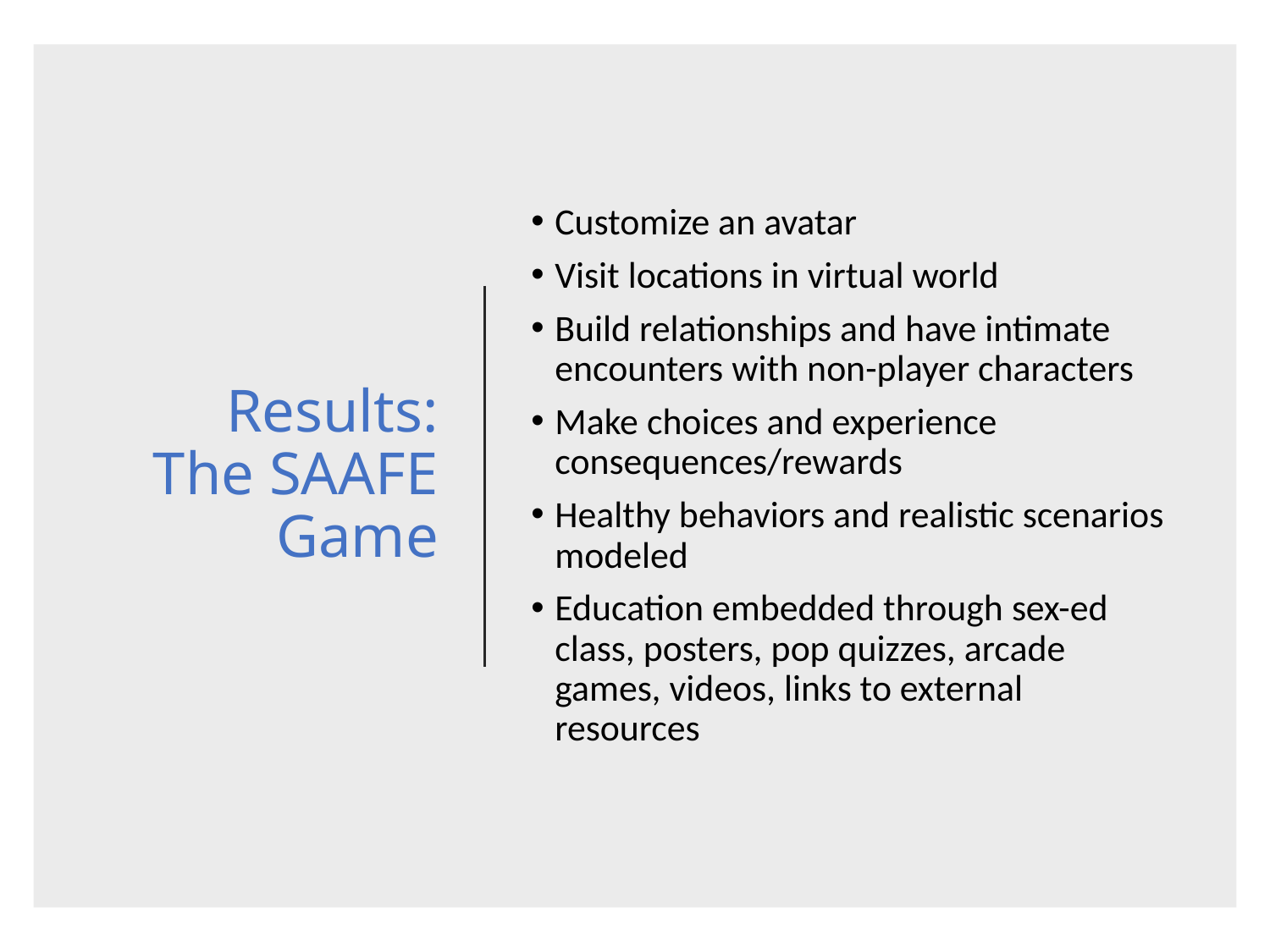

# Results: The SAAFE Game
Customize an avatar
Visit locations in virtual world
Build relationships and have intimate encounters with non-player characters
Make choices and experience consequences/rewards
Healthy behaviors and realistic scenarios modeled
Education embedded through sex-ed class, posters, pop quizzes, arcade games, videos, links to external resources

## Slide 9
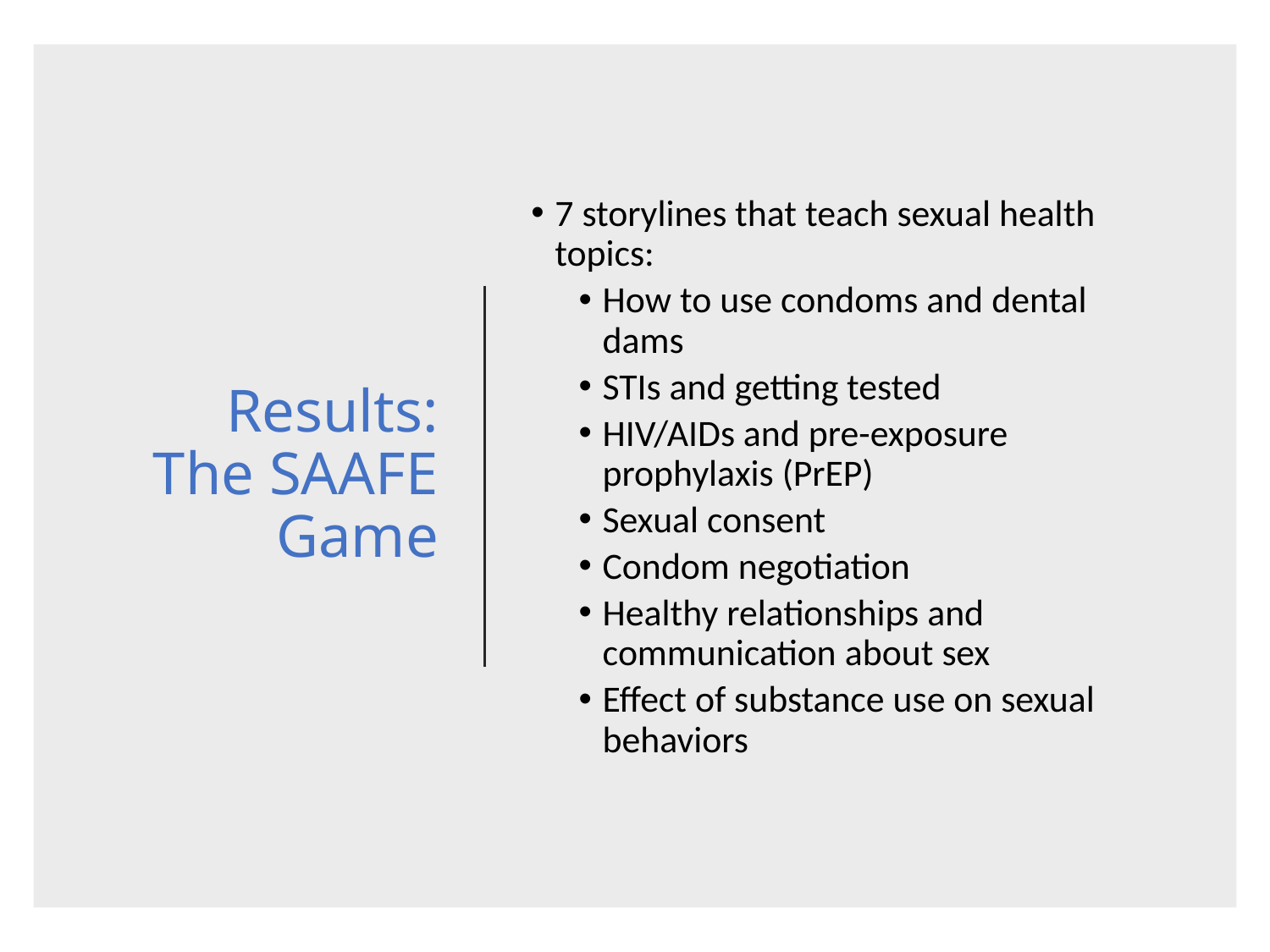

# Results: The SAAFE Game
7 storylines that teach sexual health topics:
How to use condoms and dental dams
STIs and getting tested
HIV/AIDs and pre-exposure prophylaxis (PrEP)
Sexual consent
Condom negotiation
Healthy relationships and communication about sex
Effect of substance use on sexual behaviors

## Slide 10
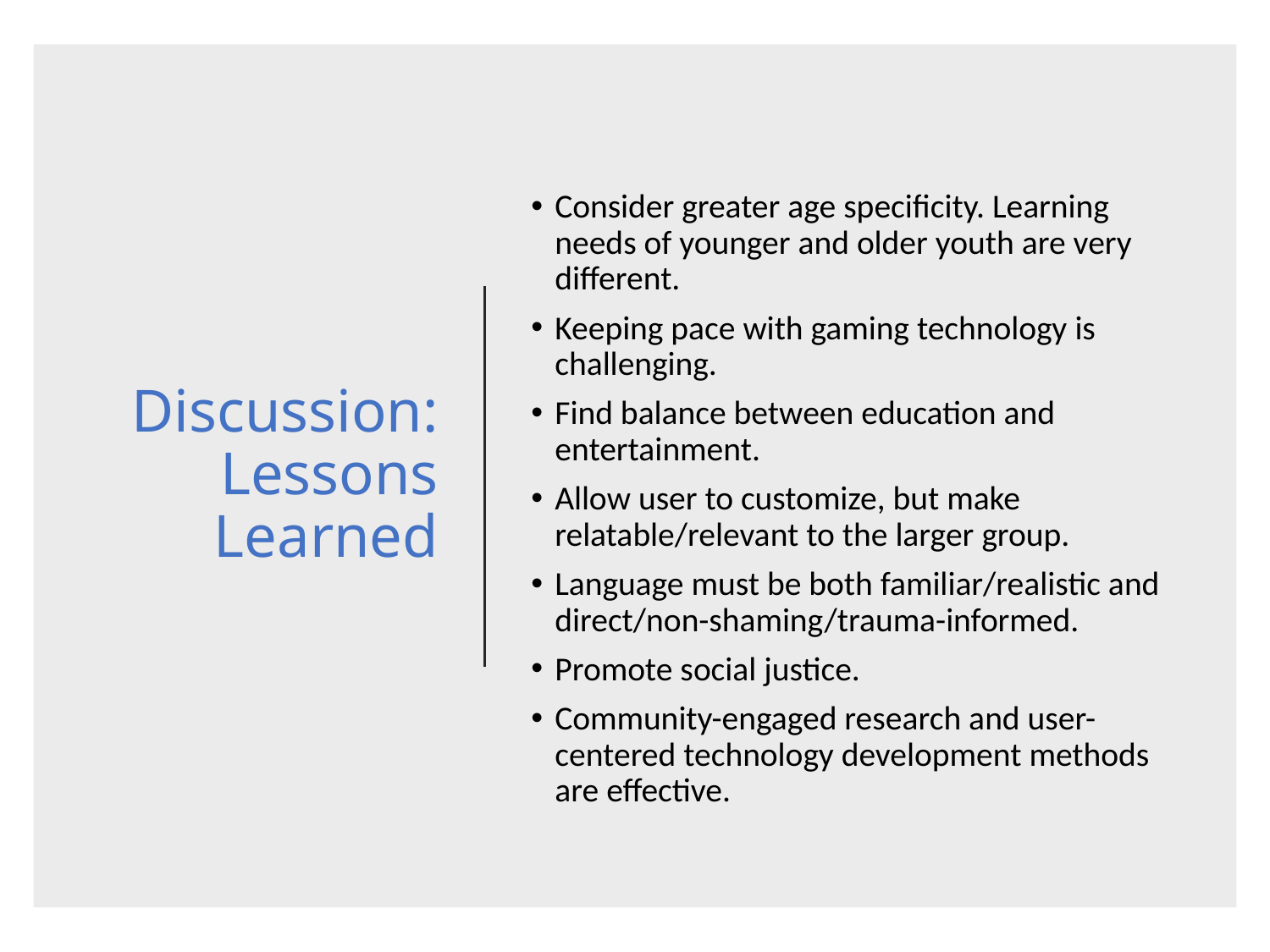

# Discussion: Lessons Learned
Consider greater age specificity. Learning needs of younger and older youth are very different.
Keeping pace with gaming technology is challenging.
Find balance between education and entertainment.
Allow user to customize, but make relatable/relevant to the larger group.
Language must be both familiar/realistic and direct/non-shaming/trauma-informed.
Promote social justice.
Community-engaged research and user-centered technology development methods are effective.

## Slide 11
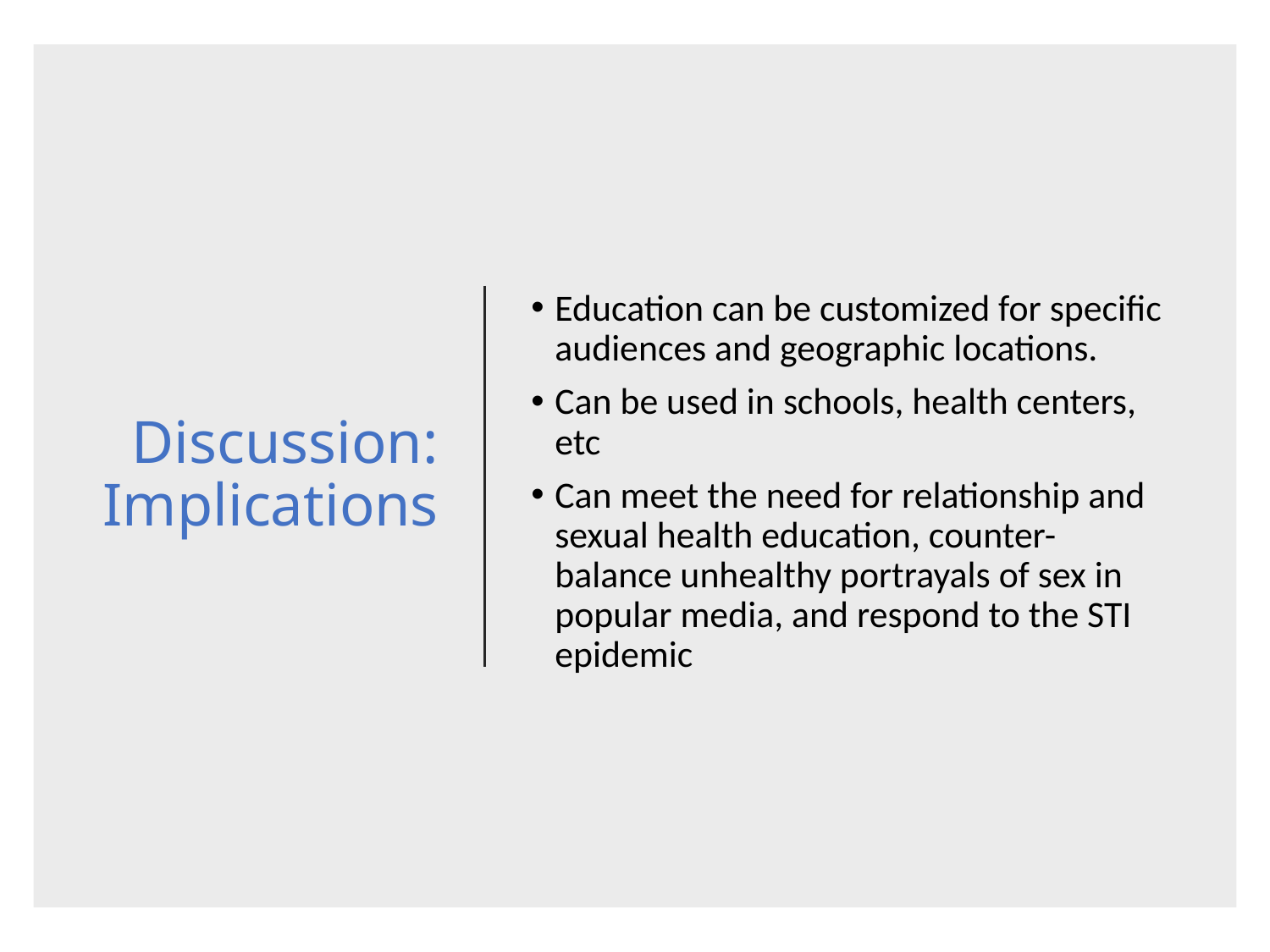

# Discussion: Implications
Education can be customized for specific audiences and geographic locations.
Can be used in schools, health centers, etc
Can meet the need for relationship and sexual health education, counter-balance unhealthy portrayals of sex in popular media, and respond to the STI epidemic

## Slide 12
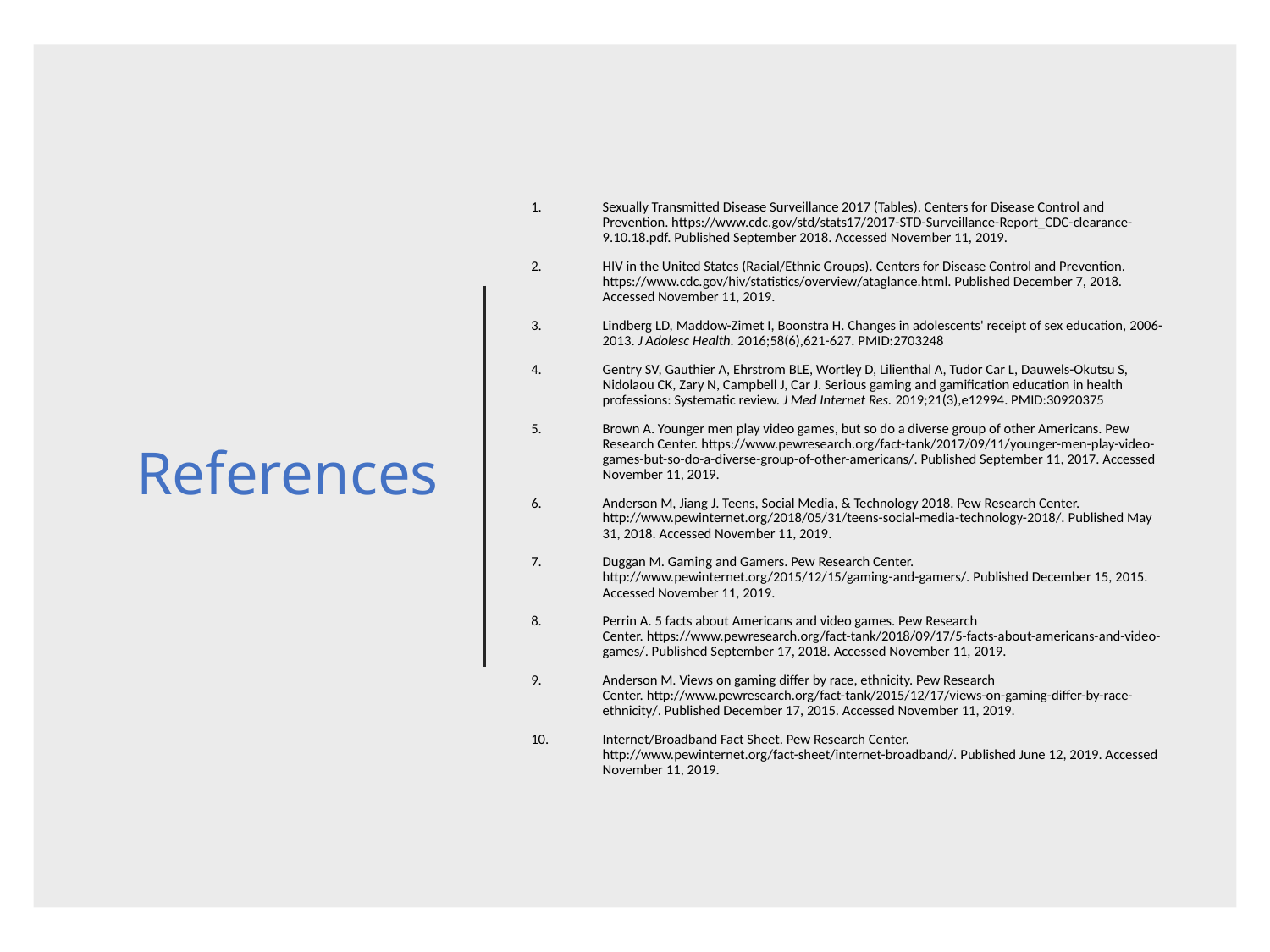

# References
Sexually Transmitted Disease Surveillance 2017 (Tables). Centers for Disease Control and Prevention. https://www.cdc.gov/std/stats17/2017-STD-Surveillance-Report_CDC-clearance-9.10.18.pdf. Published September 2018. Accessed November 11, 2019.
HIV in the United States (Racial/Ethnic Groups). Centers for Disease Control and Prevention. https://www.cdc.gov/hiv/statistics/overview/ataglance.html. Published December 7, 2018. Accessed November 11, 2019.
Lindberg LD, Maddow-Zimet I, Boonstra H. Changes in adolescents' receipt of sex education, 2006-2013. J Adolesc Health. 2016;58(6),621-627. PMID:2703248
Gentry SV, Gauthier A, Ehrstrom BLE, Wortley D, Lilienthal A, Tudor Car L, Dauwels-Okutsu S, Nidolaou CK, Zary N, Campbell J, Car J. Serious gaming and gamification education in health professions: Systematic review. J Med Internet Res. 2019;21(3),e12994. PMID:30920375
Brown A. Younger men play video games, but so do a diverse group of other Americans. Pew Research Center. https://www.pewresearch.org/fact-tank/2017/09/11/younger-men-play-video-games-but-so-do-a-diverse-group-of-other-americans/. Published September 11, 2017. Accessed November 11, 2019.
Anderson M, Jiang J. Teens, Social Media, & Technology 2018. Pew Research Center. http://www.pewinternet.org/2018/05/31/teens-social-media-technology-2018/. Published May 31, 2018. Accessed November 11, 2019.
Duggan M. Gaming and Gamers. Pew Research Center. http://www.pewinternet.org/2015/12/15/gaming-and-gamers/. Published December 15, 2015. Accessed November 11, 2019.
Perrin A. 5 facts about Americans and video games. Pew Research Center. https://www.pewresearch.org/fact-tank/2018/09/17/5-facts-about-americans-and-video-games/. Published September 17, 2018. Accessed November 11, 2019.
Anderson M. Views on gaming differ by race, ethnicity. Pew Research Center. http://www.pewresearch.org/fact-tank/2015/12/17/views-on-gaming-differ-by-race-ethnicity/. Published December 17, 2015. Accessed November 11, 2019.
Internet/Broadband Fact Sheet. Pew Research Center. http://www.pewinternet.org/fact-sheet/internet-broadband/. Published June 12, 2019. Accessed November 11, 2019.

## Slide 13
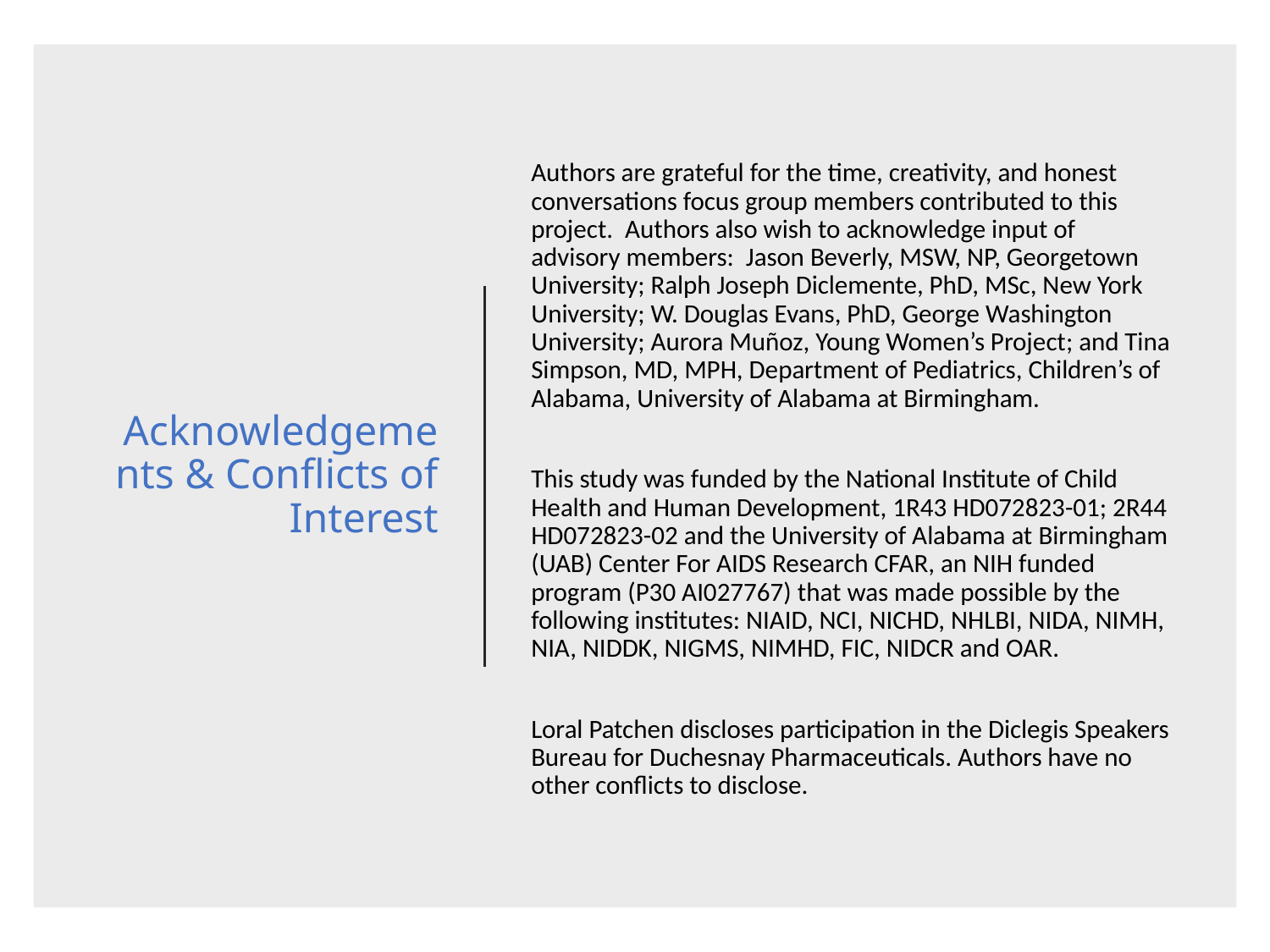

# Acknowledgements & Conflicts of Interest
Authors are grateful for the time, creativity, and honest conversations focus group members contributed to this project. Authors also wish to acknowledge input of advisory members: Jason Beverly, MSW, NP, Georgetown University; Ralph Joseph Diclemente, PhD, MSc, New York University; W. Douglas Evans, PhD, George Washington University; Aurora Muñoz, Young Women’s Project; and Tina Simpson, MD, MPH, Department of Pediatrics, Children’s of Alabama, University of Alabama at Birmingham.
This study was funded by the National Institute of Child Health and Human Development, 1R43 HD072823-01; 2R44 HD072823-02 and the University of Alabama at Birmingham (UAB) Center For AIDS Research CFAR, an NIH funded program (P30 AI027767) that was made possible by the following institutes: NIAID, NCI, NICHD, NHLBI, NIDA, NIMH, NIA, NIDDK, NIGMS, NIMHD, FIC, NIDCR and OAR.
Loral Patchen discloses participation in the Diclegis Speakers Bureau for Duchesnay Pharmaceuticals. Authors have no other conflicts to disclose.

## Slide 14
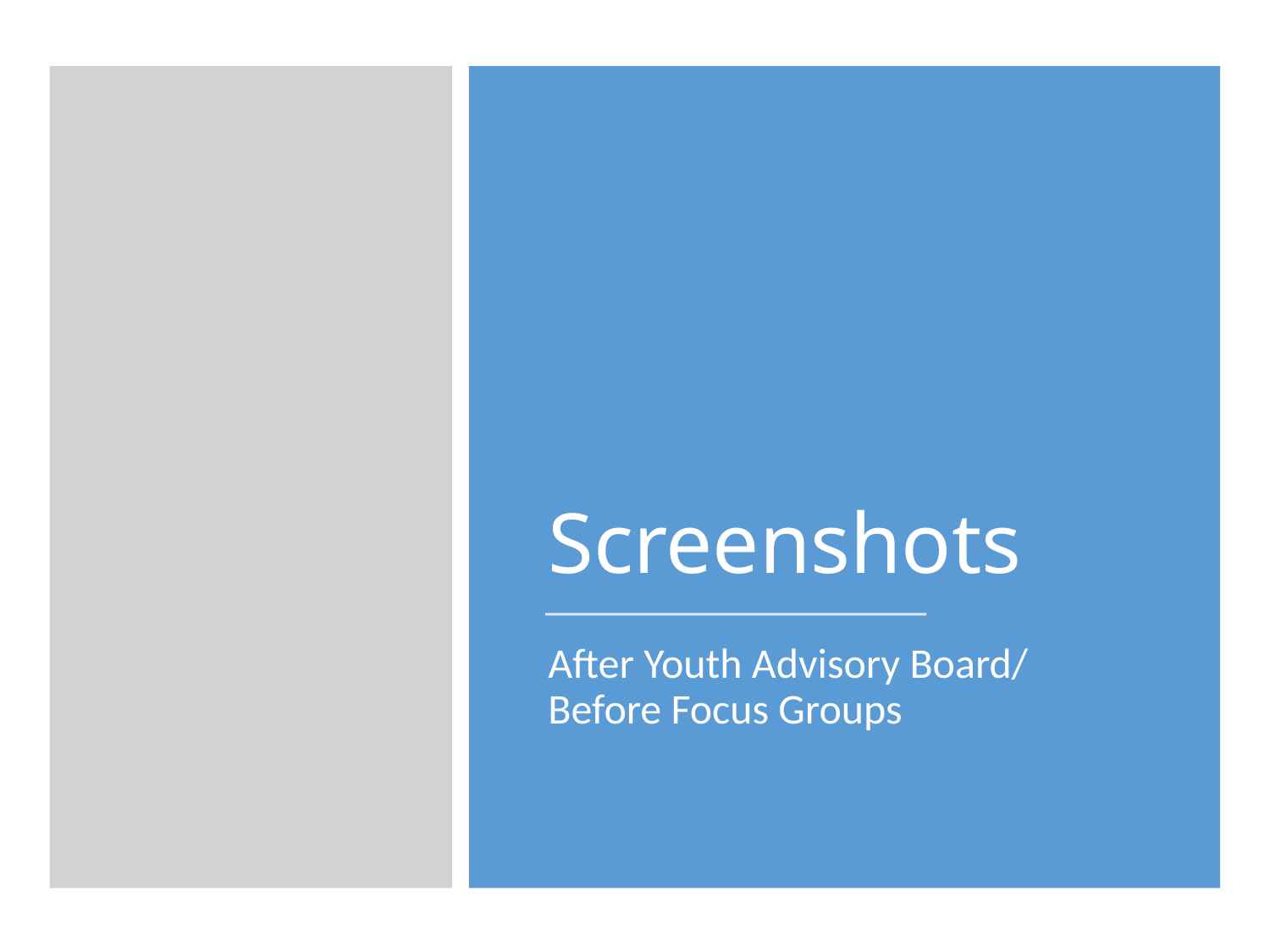

# Screenshots
After Youth Advisory Board/ Before Focus Groups

## Slide 15
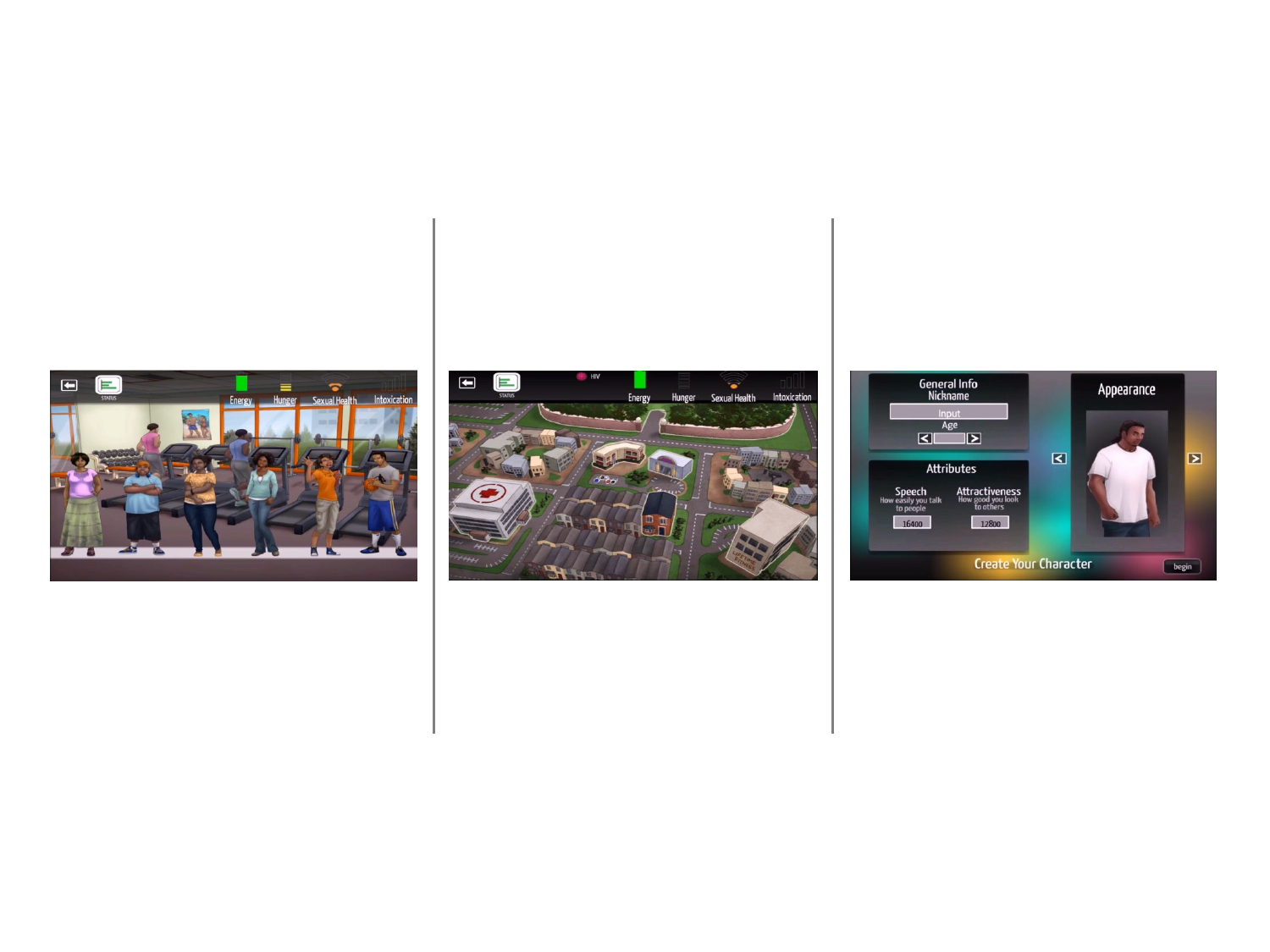

## Slide 16
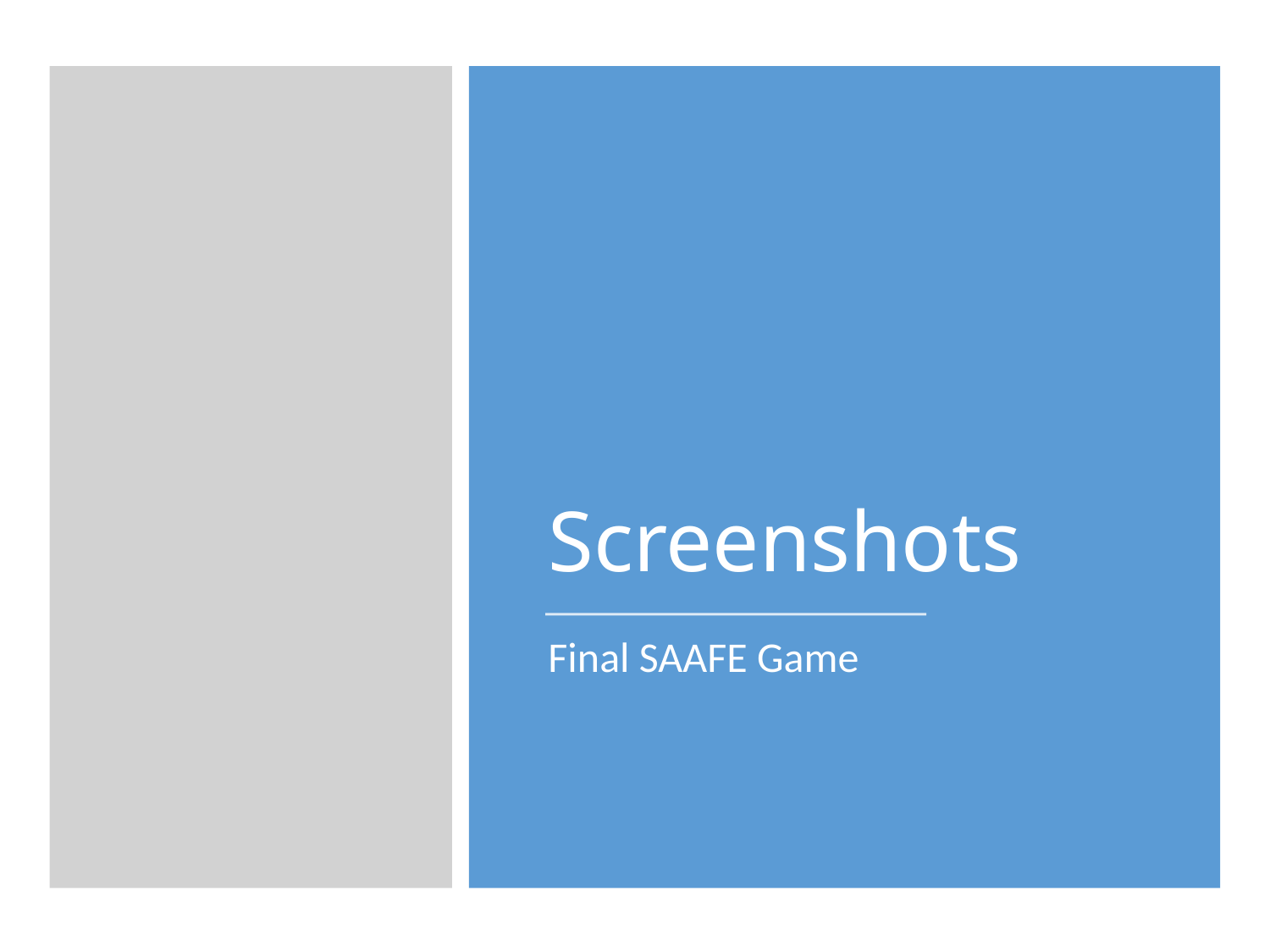

# Screenshots
Final SAAFE Game

## Slide 17
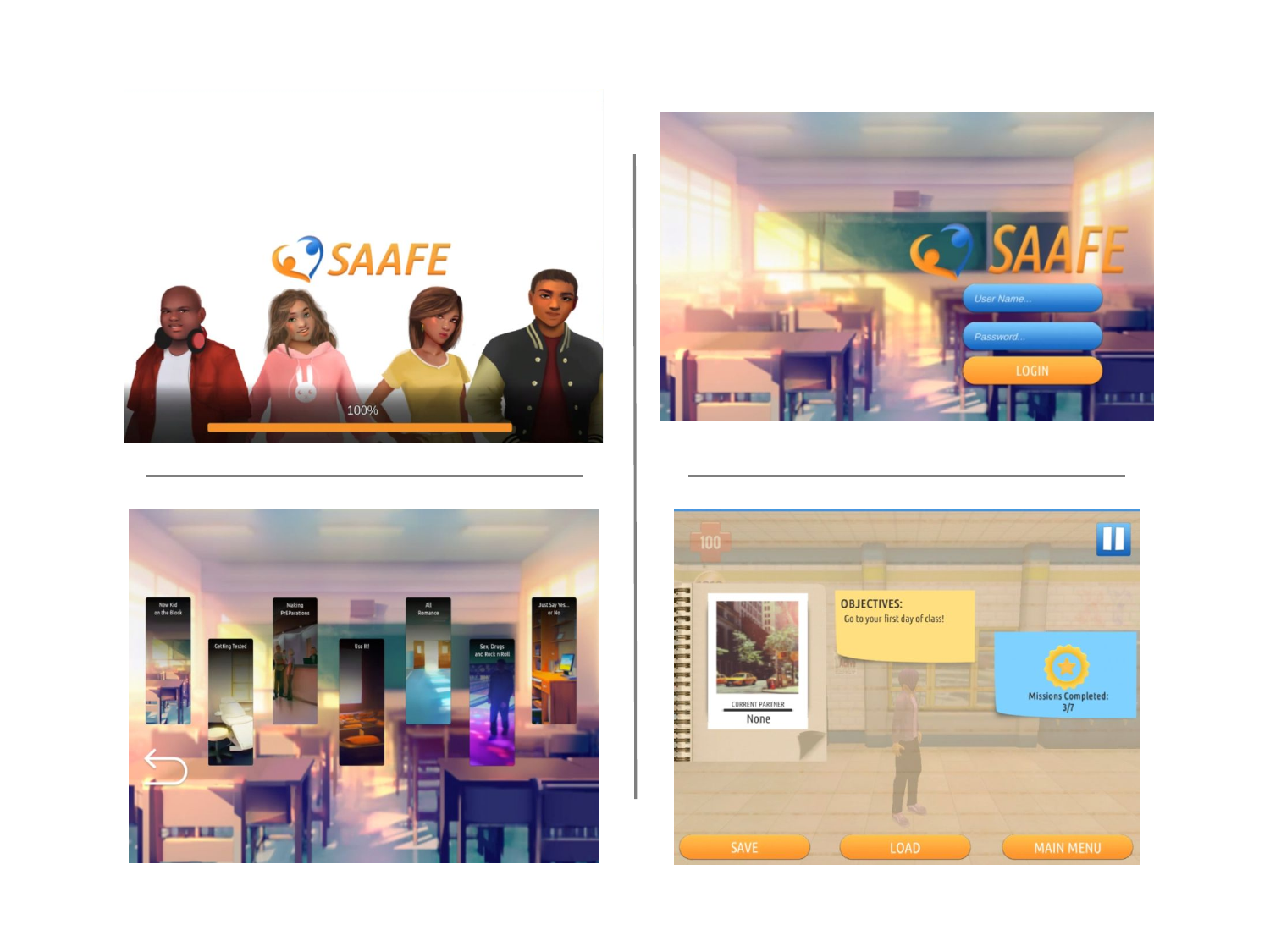

## Slide 18
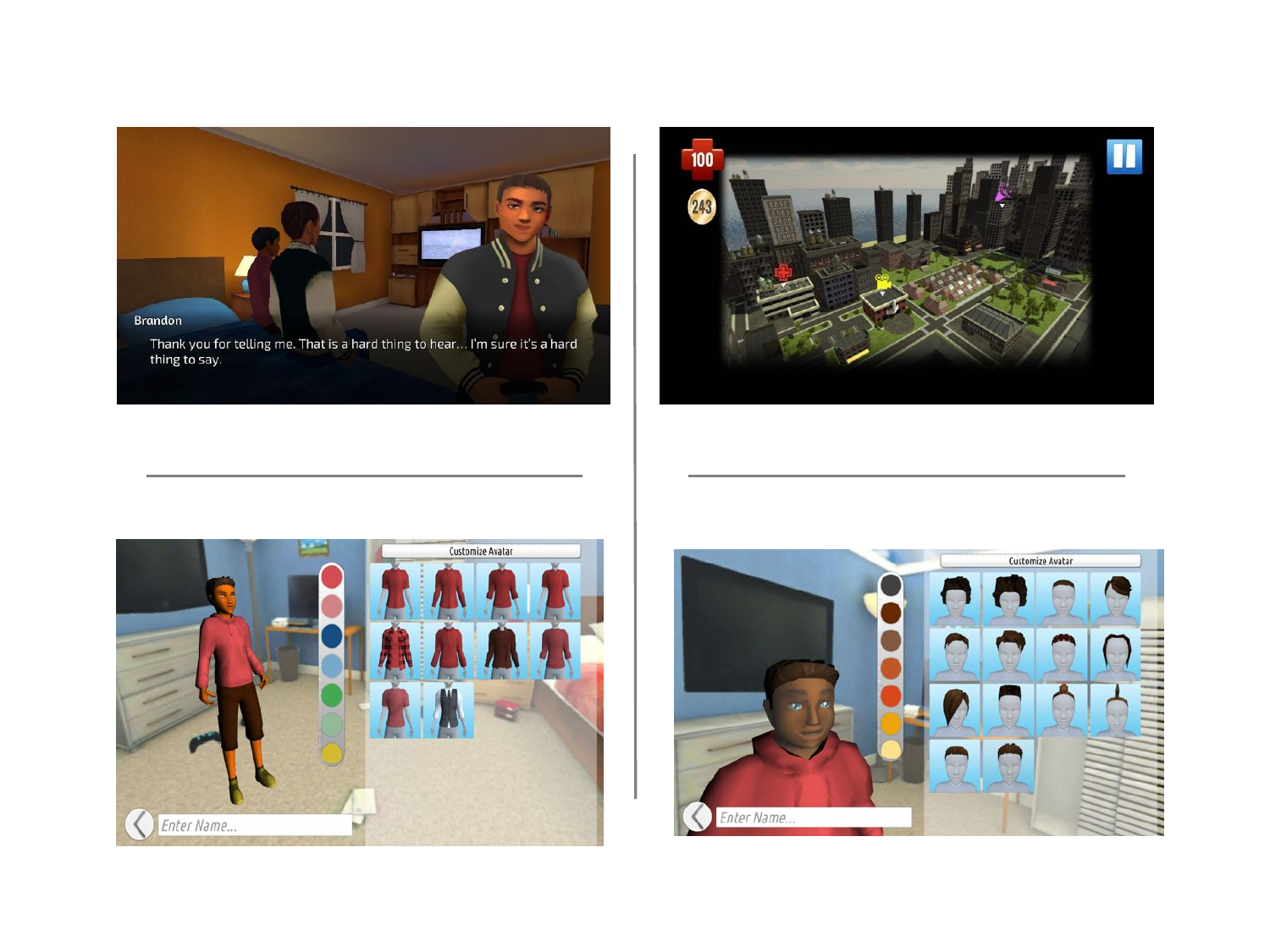

## Slide 19
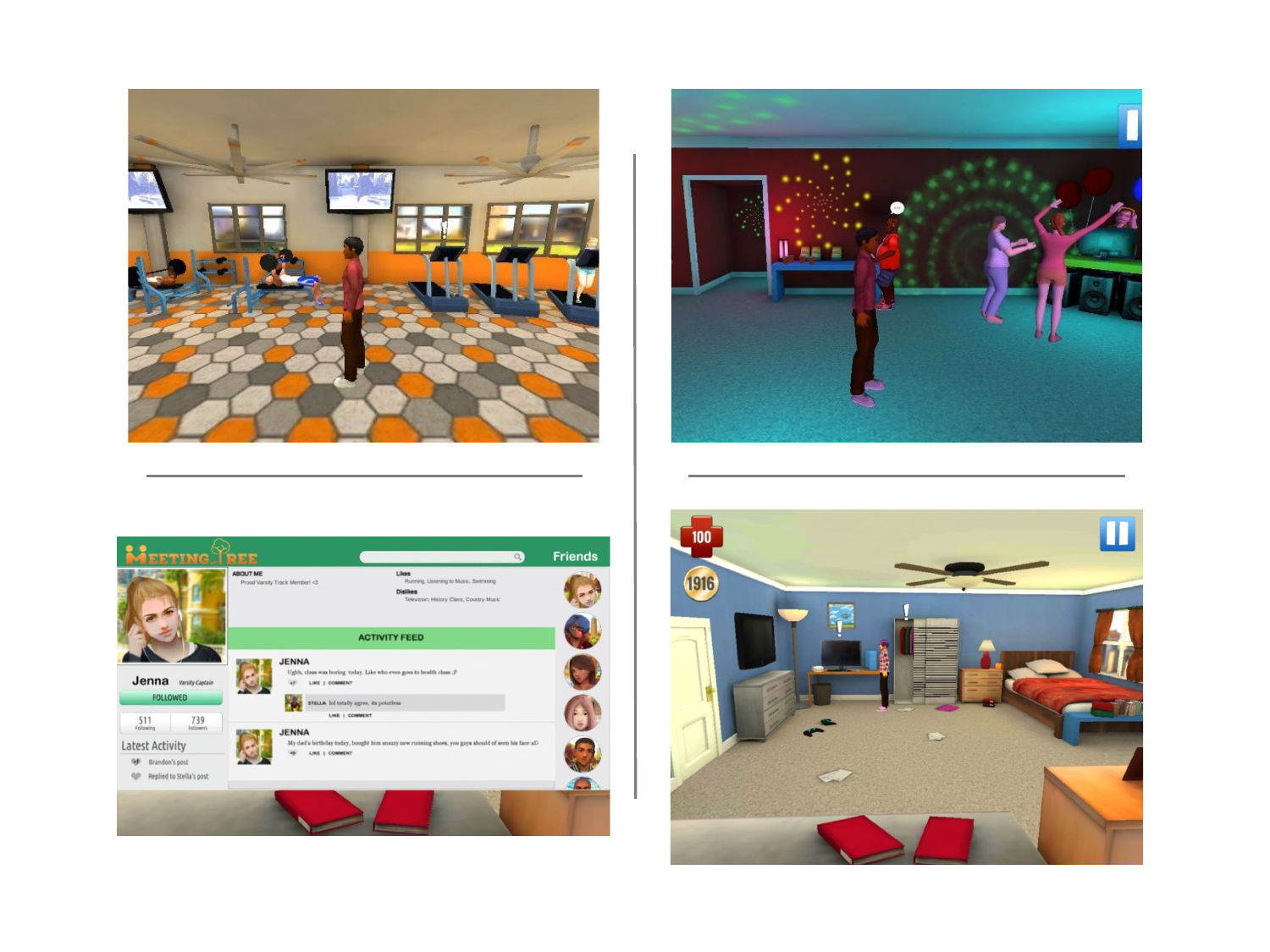

## Slide 20
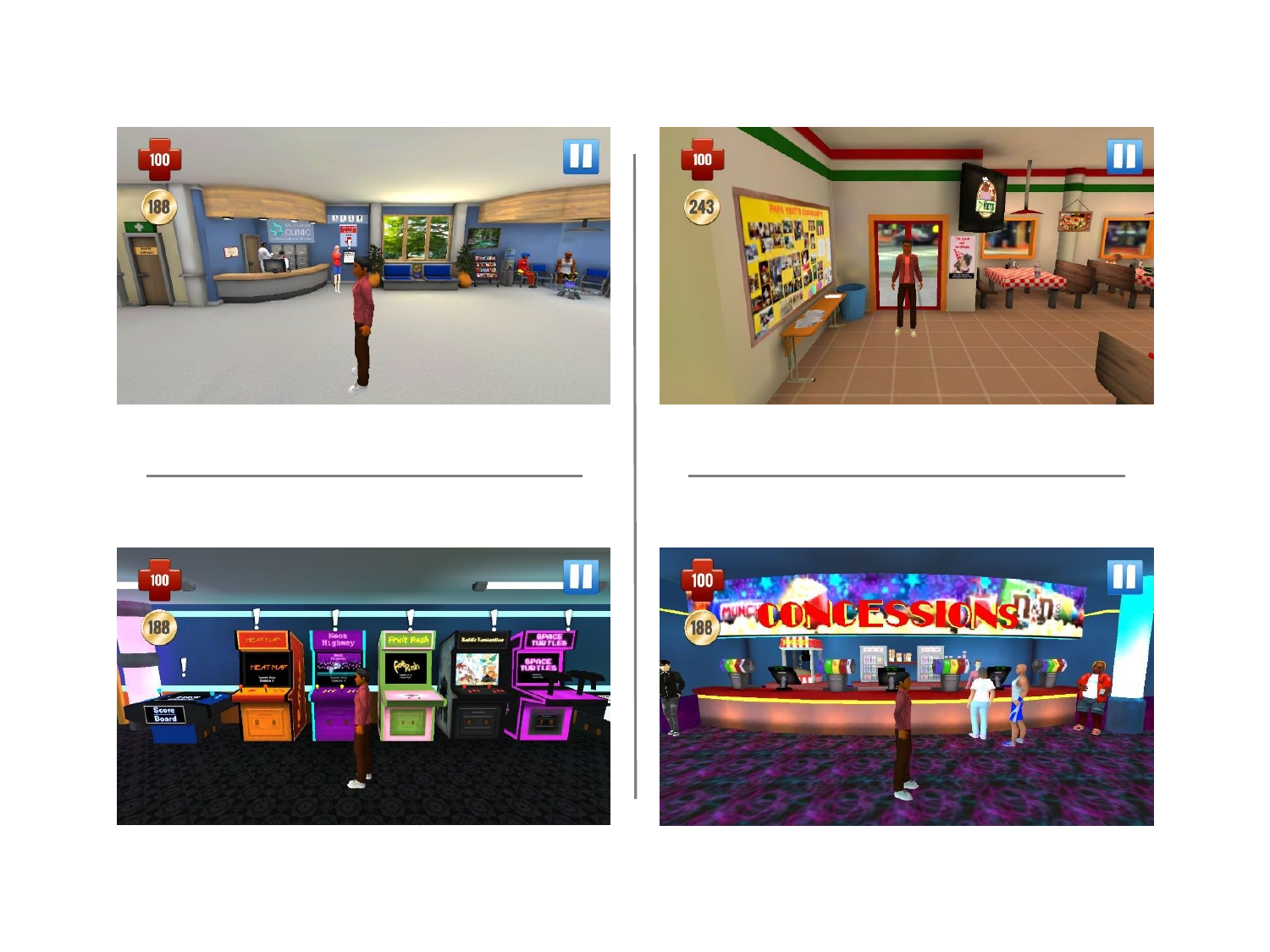

## Slide 21
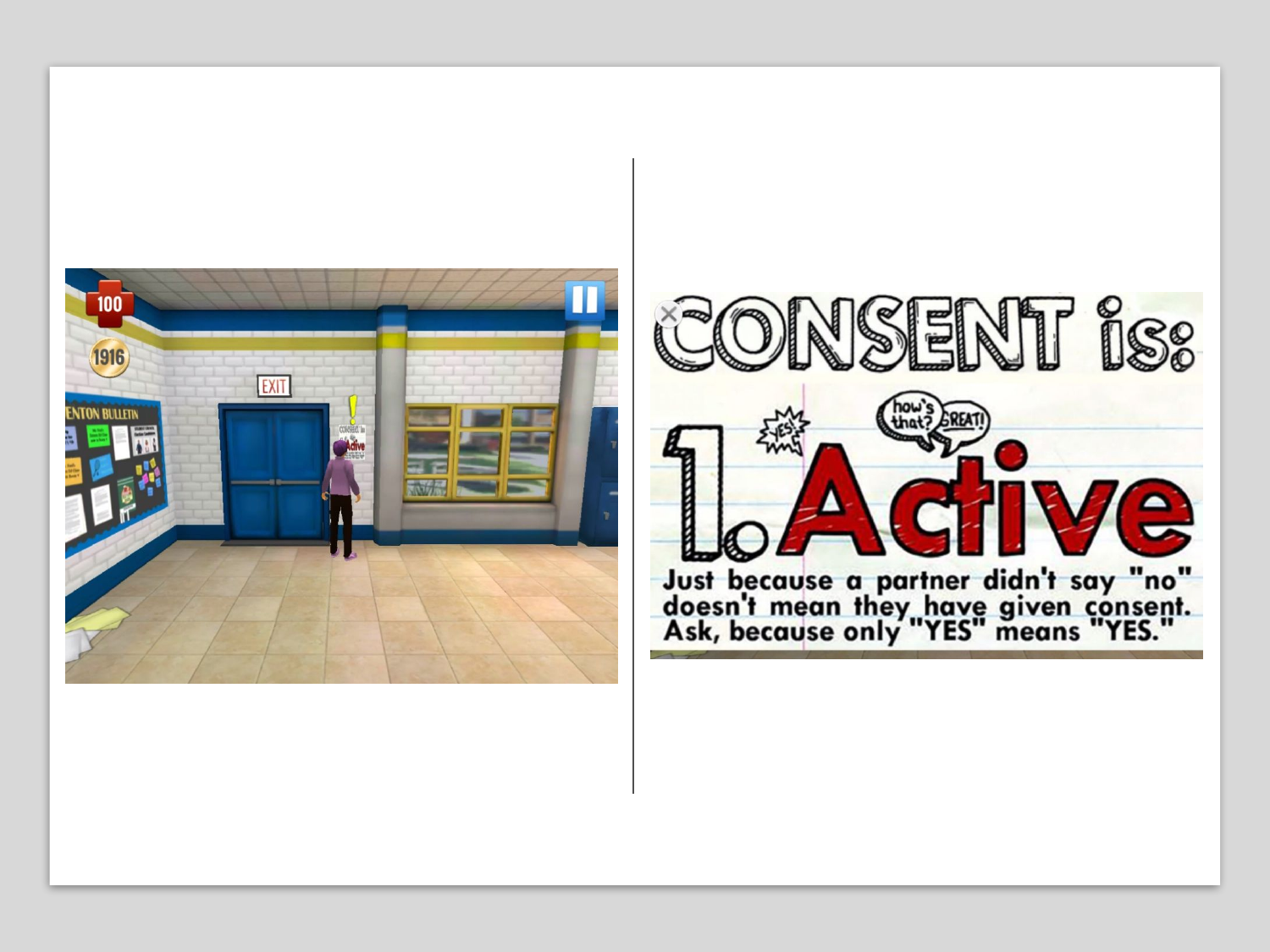

## Slide 22
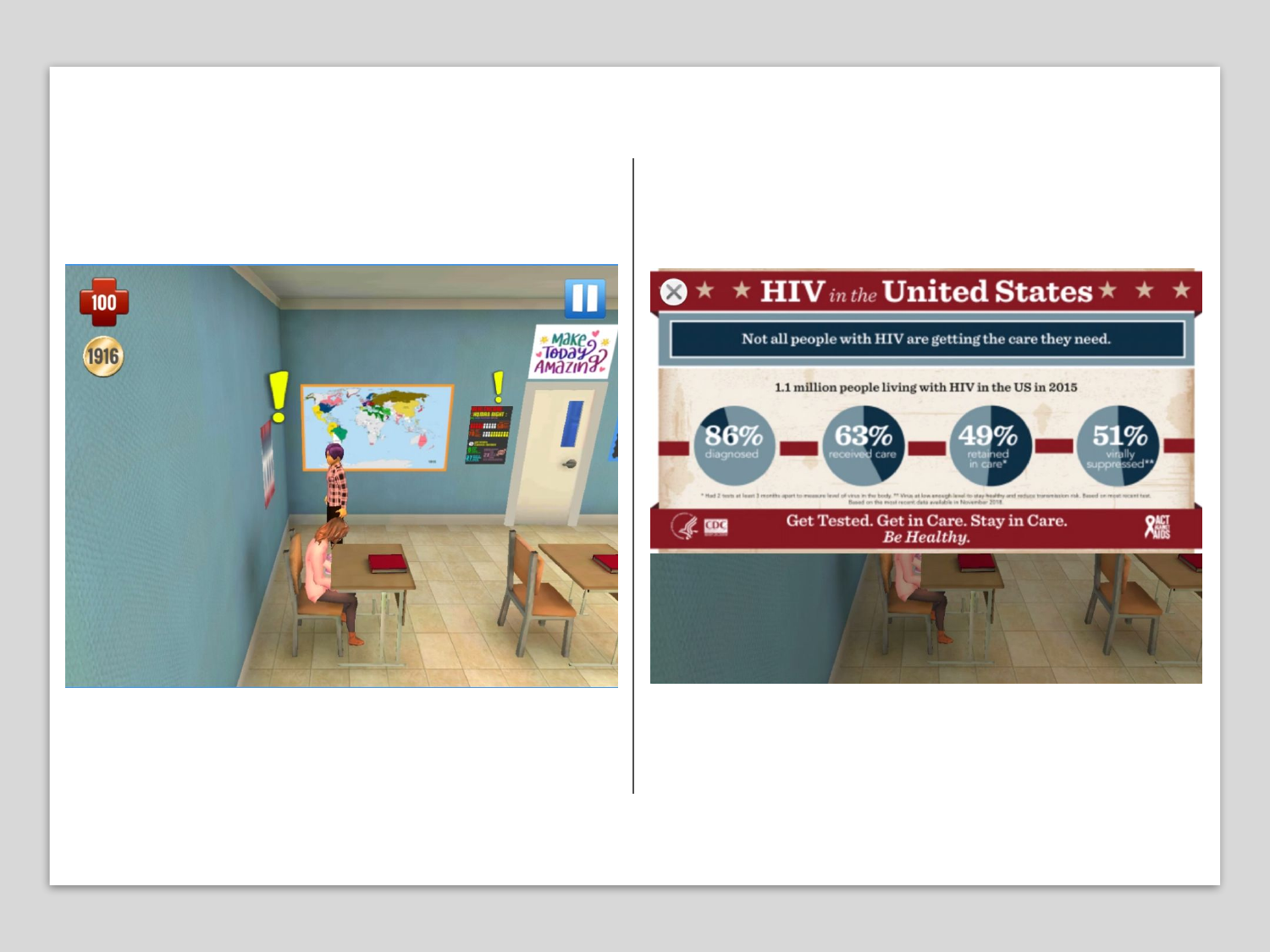

## Slide 23
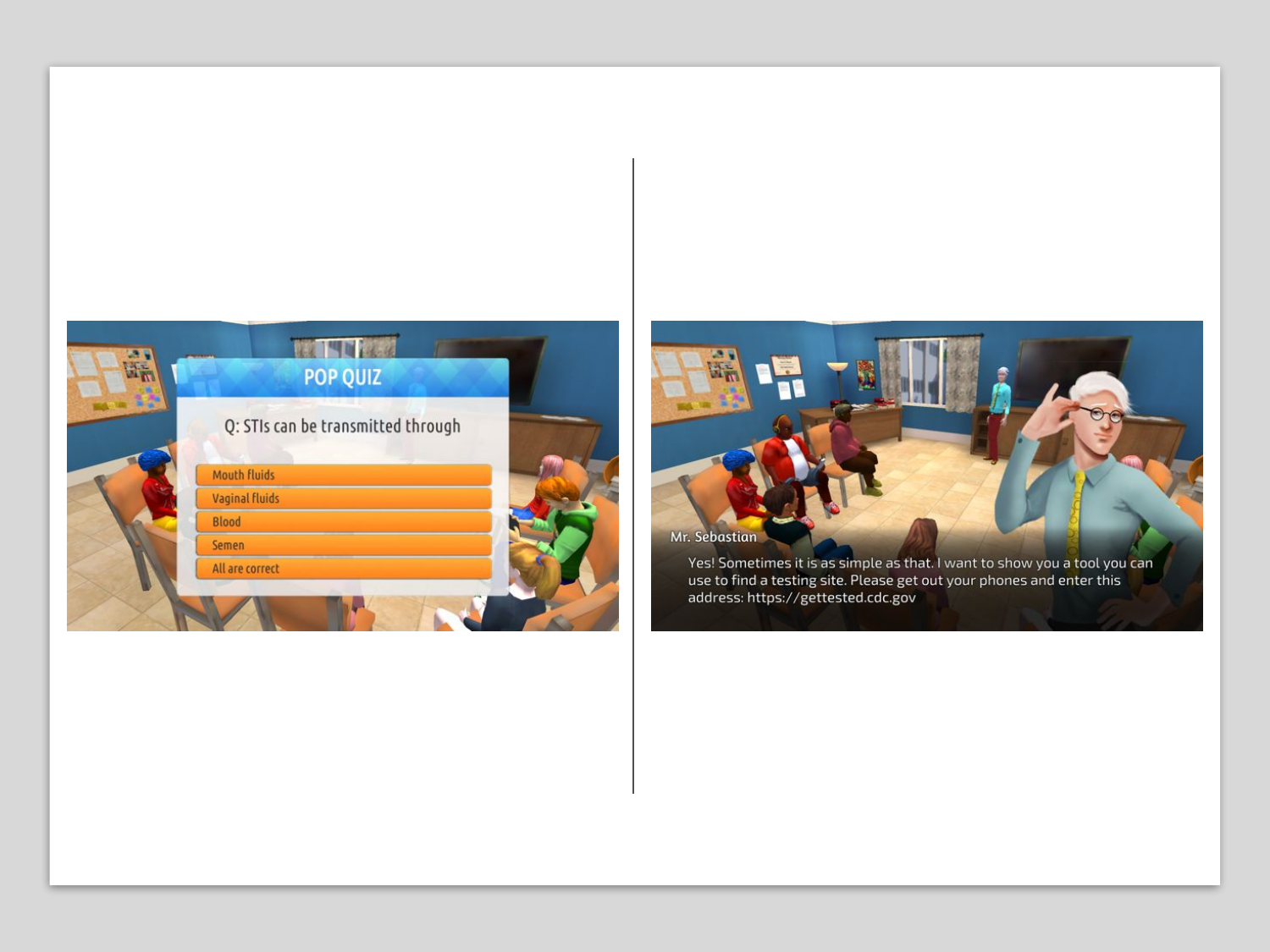

Supplement: Multimedia Appendix 1 [file games_v8i1e16254_app1.pptx]
